# Identification of the interacting proteins of *Bambusa pervariabilis × Dendrocalamopsis grandis* in response to the transcription factor *ApCtf1β* in *Arthrinium phaeospermum*

**DOI:** 10.3389/fpls.2022.991077

**Published:** 2022-09-15

**Authors:** Peng Yan, Jiawen Yu, Xinmei Fang, Shuying Li, Shan Han, Tiantian Lin, Yinggao Liu, Chunlin Yang, Fang He, Tianhui Zhu, Shujiang Li

**Affiliations:** ^1^College of Forestry, Sichuan Agricultural University, Chengdu, China; ^2^National Forestry and Grassland Administration Key Laboratory of Forest Resources Conservation and Ecological Safety on the Upper Reaches of the Yangtze River, Chengdu, China

**Keywords:** *Arthrinium phaeospermum*, *ApCtf1β*, Y2H, BiFC, GST pull-down, qRT-PCR, interaction protein

## Abstract

*Arthrinium phaeospermum* is the main pathogen that causes *Bambusa pervariabilis × Dendrocalamopsis grandis* blight. It secretes the cutinase transcription factor *ApCtf1β*, which has been shown to play an important role in *B. pervariabilis × D. grandis* virulence. However, knowledge about the interaction target genes of *ApCtf1β* in *B. pervariabilis × D. grandis* remains limited. A cDNA library for the yeast two-hybrid system was constructed from *B. pervariabilis × D. grandis* shoots after 168 h treatment with *A. phaeospermum*. The library was identified as 1.20 × 10^7^ cfu, with an average insert >1,000 bp in size and a 100% positive rate, providing a database for the subsequent molecular study of the interaction between *A. phaeospermum* and *B. pervariabilis × D. grandis*. The yeast two-hybrid (Y2H), bimolecular fluorescence complementation (BiFC), and glutathione-S-transferase (GST) pull-down assays were used to screen for and identify two *ApCtf1β* interacting target proteins, *BDUbc* and *BDSKL1*, providing a reliable theoretical basis to study the molecular mechanism underlying *B. pervariabilis × D. grandis* resistance in response to *A. phaeospermum*, which would, in turn, establish a platform to develop new strategies for the sustainable and effective control of the blight diseases of forest trees.

## Introduction

Hybrid bamboo (*Bambusa pervariabilis × Dendrocalamopsis grandis*) is widely used as greenery, paper making and bamboo weaving to produce bamboo shoots and as an ornamental. It has also been introduced in the Yangtze River basin to build ecological barriers (Zhao et al., 2004; Li et al., 2020). An infestation of *A. phaeospermum* has recently caused extensive dieback of hybrid bamboos (Zhu et al., 2009). Our previous studies were mainly focused on investigating the physiology of disease development and biological characteristics of pathogenic fungi, determining the virulence of pathogenic toxin proteins, isolating and screening antagonistic fungi, as well as the hybrid bamboo blight fungus genome and the transcriptome, proteome, and metabolome of hybrid bamboo induced by pathogen infestation and toxin proteins (Li and Zhu, 2012; Li et al., 2013a,b). However, the molecular mechanism by which *Bambusa pervariabilis* × *Dendrocalamopsis grandis* responds to *A. phaeospermum* is not yet understood.

Pathogenic fungi can cause a wide range of plant diseases. Their hyphae can directly penetrate the plant’s epidermal cells or form special infestation structures (e.g., adherent spores) that invade the plant to extract nutrients. Plants have evolved an elaborate, cell-based immune system with innate and induced defenses to escape pathogenic fungi (Dangl and Jones, 2001; Ausubel, 2005; Jones and Dangl, 2006; Dodds and Rathjen, 2010). The earliest known mechanism of plant-pathogen interactions was the gene-for-gene hypothesis proposed by Flor in the mid-twentieth century (Flor, 1942). Following advances in research and accumulated experimental evidence, the zigzag model is now widely recognized and accepted by the academic community (Jones and Dangl, 2006). The study of the molecular mechanism by which pathogenic fungi infest hosts is centered on the functional regulatory network of pathogenic key genes. The key genes related to the development of the pathogenic fungus infesting structure and pathogenicity are explored using gene knockout and backfill overexpression technologies and phenotype analysis of mutants. The cloning and functional study of key pathogenicity genes interacting within the plant immune system can help resolve the host molecular resistance mechanism (Zhang et al., 2015). Therefore, it is essential to screen for and identify target proteins involved in fungi-host interactions and clarify the interaction mechanism between target protein function and pathogenic effector genes to resolve the molecular mechanisms underlying fungal pathogenicity and plant disease resistance. The yeast two-hybrid (Y2H) assay has been widely used to determine protein–protein interactions in organisms (Chen et al., 2012). Furthermore, constructing cDNA libraries is a popular methodology in molecular biology. By combining cDNA libraries and yeast two-hybrid technology, we can study the interactions between known proteins and target proteins more conveniently, which is important for studying function-specific proteins. Currently, the yeast two-hybrid system has been used to screen for target proteins that interact with pathogenic fungi on host plants such as rice (*Oryza sativa* L.; Singh et al., 2014), Arabidopsis (*Arabidopsis thaliana*; Napoli et al., 2021), potato (*Solanum tuberosum* L.; McLellan et al., 2021), rubber tree [*Hevea brasiliensis* (Willd. ex A. Juss.) Muell. Arg.; Song et al., 2022], and sweet potato [*Dioscorea esculenta* (Lour.) Burkill; Zhang et al., 2020]. Constructing a high-quality yeast two-hybrid library is necessary to achieve efficient and high-quality screening of reciprocal target proteins.

The cuticle is a biopolyester on the surface of higher plants, whose main roles include controlling the water exchange between plants and the environment, regulating plant body temperature, preventing mechanical damage, and defending against pathogens. The cuticle is the first barrier against pathogenic infestation. The cuticle also involved in signal production and transmission during plant development and plant-pathogen interactions. The study of cutinase originated from the search for the mechanism of plant pathogens, where pathogenic fungus secrete cutinase to break down the cuticle when invading the plant epidermis (Li et al., 2002; Wang et al., 2016), invade the interior, and eventually affect plant growth. Transcriptional activation of cutinase genes in both *Fusarium solani* and *Aspergillus nidulans* is mediated by the cutinase transcription factor CTF1 (Li and Kolattukudy, 1997; Li et al., 2002). In *Fusarium solani* f. sp. *pisi* (Rogers et al., 1994), *Curvularia lunata* (Liu et al., 2016), and other plant pathogens, deletion of the gene encoding cutinase reduces virulence or eliminates pathogenicity to the host plant (Davies et al., 2000; Li et al., 2003; Zhang et al., 2005; Skamnioti and Gurr, 2007; Marino et al., 2012; Liu et al., 2016; Wang et al., 2016). However, in other studies, cutinase knockout mutants did not lose pathogenicity (Chasan, 1992; Stahl and Schäfer, 1992; Sweigard et al., 1992).To date, several studies have demonstrated that cutinases are also associated with the triggering of host-derived signals, fungal spore attachment, and carbon acquisition during saprophytic growth (Köller, 1991; Deising et al., 1992; Kolattukudy et al., 1995; Skamnioti and Gurr, 2007; Cheung et al., 2009; Järvinen et al., 2009; Auyong, 2015). Cutinases are essential for spore adhesion in *Uromyces viciae-fabae* and *Colletotrichum graminicola* (Deising et al., 1992; Pascholati et al., 1992) and may also play a role in inducing defense responses (Parker and Köller, 1998; Zhang et al., 2014). For example, in *Sclerotinia sclerotiorum*, *SsCut* acts as a PAMP to induce defense responses in several host plants. Degradation of the plant epidermis by cutinases secreted by fungal pathogens may lead to the release of DAMPs (De Lorenzo et al., 2011).

In our previous study, the cutinase transcription factor *ApCtf1β* was confirmed to be the key virulence factor of *A. phaeospermum* in the stress response of *B. pervariabilis × D. grandis* (Fang et al., 2021). However, few studies have investigated the screening and functions of cutinase transcription factors in host plants, especially in *B. pervariabilis × D. grandis*, thus severely limiting our understanding of the pathogenesis of *A. phaeospermum* and the molecular mechanism of the response of bamboo to its infection. In the present study, we constructed a yeast library of hybrid bamboo infested with *A. phaeospermum* and screened the *ApCtf1β* reciprocal protein using a yeast two-hybrid assay. Verification was *via* GST pull-down and fluorescent bimolecular complementation assays. The findings of this study will provide a reliable theoretical basis for the study of the *A. phaeospermum* pathogenic pathway and the molecular mechanism underlying *B. pervariabilis × D. grandis* resistance in response to pathogenic infection, establishing a platform for developing new strategies for sustainable and effective control of wilting blight diseases in forest trees.

## Materials and methods

### Materials

#### Microorganism

*Arthrinium phaeospermum* was isolated from diseased *B. pervariabilis × D. grandis* using the tissue isolation method. The accession number for *A. phaeospermum* whole genome information in the GenBank database is QYRS0000000.1. The isolate was stored at the China Forestry Culture Collection Center (reference number: cfcc 86,860; http://www.cfcc-caf.org.cn/, Accessed on April 6, 2007).

#### Plant tissue sample

One-year-old *B. pervariabilis × D. grandis* (bamboo) seedlings purchased from Shuyang Qichen Bamboo Seedling Co., Ltd. (Suqian, China) and *Nicotiana benthamiana* (tobacco) seedlings purchased from Huayueyang Biotech Co., Ltd. (Beijing, China) were planted in the greenhouse of Sichuan Agricultural University, Chengdu, Sichuan, China at a temperature of 25°C and humidity of 60%. The accession number for dual-seq of *B. pervariabilis × D. grandis* infected by *A.phaeospermum* in the NCBI database is SAMN19312317.

### Methods

#### Construction of a yeast library of *Bambusa pervariabili*s × *Dendrocalamopsis grandis* infected by *Arthrinium phaeospermum*

##### RNA extraction and reverse transcription

*Arthrinium phaeospermum* was inoculated onto PDA (containing 200 g of potato, 20 g of glucose, and 20 g of agar per liter) and incubated at 25°C for 5 days. Small wounds were created at the shoot nodes of *B. pervariabilis × D. grandis via* needle pricking, inoculated with a 5-mm fungal cake, and covered with film to retain moisture. Total RNA was extracted using TRIzol (Beijing TransGen Biotech Co., Ltd., China) after 168 h of *A. phaeospermum* treatment. Residual DNA was removed using DNase I digestion enzyme, and total RNA concentration and purity were determined using a spectrophotometer. RNA integrity was detected *via* 1% agarose gel electrophoresis—a 28S:18S ratio of 2:1 indicated suitable RNA integrity, and an A260/A280 ratio between 1.8 and 2.0 indicated suitable RNA purity. mRNA was isolated and purified using the Oligotex mRNA Midi Kit(QIAGEN-CN, Shanghai, China). The quality and concentration of mRNA were detected using 1% agarose gel and a spectrophotometer (NANODROP, ThermoFisher Scientific-CN, Shanghai, China), respectively.

##### Primary library (uncut type) construction and preservation

mRNA obtained from the previous step was reverse-transcribed to obtain the first strand of cDNA using the CloneMiner II cDNA library construction kit according to the manufacturer’s instructions. Next, first-strand cDNA was used as the PCR template to synthesize the second strand of cDNA. The cDNA was ligated with three frames of attB recombinant connectors (one copy of each of the three connectors), separated, and collected. Approximately 500–4,000 bp of double-stranded cDNA was collected and ligated into the vector *via* the BP recombination reaction. The recombination products were electrotransformed into *Escherichia coli* DH10B receptor cells, and the cDNA library was obtained by culturing *E. coli* in a shaking incubator. After 1-h incubation, 10 μl of the transformed *E. coli* stock solution was diluted 100 times, and 50 μl of the diluted suspension was aspirated and coated with LB medium (50 mg/L Amp) to identify library capacity. Twenty-four single clones were randomly selected from the colonies grown on the medium and identified *via* colony PCR. Their insert fragment length and recombination rate were analyzed. The remaining bacterial broth was mixed with sterile glycerol to a final concentration of 20% and stored in liquid nitrogen at −80°C to be used as the primary library broth. Library volume identification was calculated as follows: cfu/ml = clones on medium/50 μl × dilution × 1 × 10^3^ μl. Total library CFU = cfu/ml × total volume of library broth (ml).

##### Secondary library construction and preservation

The validated primary library was added to the corresponding resistant LB medium and incubated overnight at 30°C in a shaker. The plasmids were extracted, their OD measured, and their presence detected *via* electrophoresis at the end of the culture period. The extracted plasmid was diluted to 300 ng/μl and mixed with the pGADT7-DEST vector for LR recombination reaction. The reaction product was electrotransformed into *E. coli* DH10B receptor cells, and the transformed product was inoculated into a new 15-ml centrifuge tube and incubated for 1 h in a shaker at 37°C and 250 r/min. The culture was coated with medium, and 24 single clones were randomly picked from the resulting colonies. The clones were identified *via* colony PCR and analyzed for insert length and recombination rate. Sterile glycerol was added to the remaining bacterial broth to a final concentration of 20%, and the mixture was stored at −80°C in liquid nitrogen to be used as the secondary library broth.

##### Yeast two-hybrid cDNA library quality and identification

The Y187 yeast strain was transformed with 5 μg of secondary library plasmid, and 100 μl of dilutions (at 1:10, 1:100, 1:1,000, and 1:10,000) were spread on 150 mm SD/−Leu mediums. The medium were inverted and incubated at 30°C until clones appeared (at 3–6 days). Transformants were collected to determine the titer of the library, and 24 clones were picked for PCR identification using the T7SP primer and 3′AD primer. Electrophoresis was performed to determine the size of inserted cDNA fragments.

#### Screening for host target proteins interacting with *ApCtf1β* using the yeast two-hybrid technique

##### Acquisition of *ApCtf1β* from *Arthrinium phaeospermum*

According to the manufacturer’s instructions, the total RNA of *A. phaeospermum* was extracted using the TransZol Up RNA Extraction Kit (TransGen). The extracted products were tested for purity and integrity *via* agarose electrophoresis and microspectrophotometry. According to the manufacturer’s instructions, RNA that met the target requirements was reverse-transcribed to synthesize cDNA for backup using the All-in-One First-Strand cDNA Synthesis Super Mix for PCR (TransGen a). The homologous primers *ApCtf1β*-F/*ApCtf1β*-R (Supplementary Table S1) were designed based on the *ApCtf1β1* and *ApCtf1β2* genes (MK789640 and MK789641) in the NCBI database. PCR amplification was performed using *A. phaeospermum* cDNA as the template. The PCR products were detected *via* 1% agarose gel electrophoresis, recovered, and ligated to the cloning vector pEASY-Blunt Zero (TransGen) to obtain the recombinant vector. The ligated products were transformed into receptor cells DH5α. Positive transformants were picked following verification *via* colony PCR and sent to Tsingke for sequencing to confirm that the target genes were not mutated.

##### Construction of the pGBKT7-*ApCtf1β* bait vector

The homologous recombinant primers of *ApCtf1β* were designed (Supplementary Table S1). The cloned plasmid successfully sequenced in the previous step was used as a template for the PCR reaction, and the PCR products were recovered. The pGBKT7 vector was recovered *via* overnight digestion at 37°C using the restriction endonuclease XmaI. The recovered *ApCtf1β* fragment was ligated with the linearized pGBKT7 vector at 50°C for 15 min using the Trelief™ SoSoo Cloning Kit (Tsingke) with homologous recombinase and transferred into *E. coli* receptor DH5α for colony PCR identification. Plasmids were extracted from the positive clones using magnetic beads (Shan et al., 2011) and sent to Tsingke for sequencing to confirm that the target genes were not mutated.

##### Validation of bait vector toxicity and self-activation

The yeast receptor state Y2HGold was prepared for the decoy vector toxicity assay, self-activation assay, and subsequent library screening experiments using the standard PEG/LiAc method (Chen et al., 2009). Plasmids containing the decoy vector (pGBKT7-*ApCtf1β*), negative control (pGBKT7), positive control (pGBKT7-53 + pGADT7-T), and negative control (pGBKT7-Lam + pGADT7-T) were also transferred into Y2H Gold receptor cells using the PEG/LiAc method. Reactions were constructed according to Table 1 and cultured at 30°C for 5–7 days. Growth was observed to determine the presence of self-activation and toxic effects.

**Table 1:** Self-activation and toxicity assay reaction constructs.

Reaction	Medium type	Existence of clone	Clone color
pGBKT7-53 + pGADT7-T	SD/−Trp/−Leu/X-α-Gal	Yes	Blue
pGBKT7-Lam + pGADT7-T	SD/−Trp/−Leu/X-α-Gal	Yes	White
pGBKT7-*ApCtf1β* + pGADT7	SD/−Trp/−Leu/X-α-Gal	Yes	White
pGBKT7-*ApCtf1β* + pGADT7	SD/−Trp/−Leu/-His/X-α-Gal	No	-
pGBKT7-*ApCtf1β* + pGADT7	SD/−Trp/−Leu/-His/−Ade/X-α-Gal/AbA	No	-

##### SOP-mating method for yeast two-hybrid library screening

The extracted pGBKT7-*ApCtf1β* plasmid was transferred into the yeast receptor cells Y2HGold. Yeast monoclonal clones that grew normally in SD/−Trp medium were selected for verification *via* PCR. Single colonies of Y2HGold yeast strain transformed with the recombinant plasmid were picked and inoculated in 50 ml of SD/−Trp liquid medium. Y2H Gold yeast solution (5 ml) transformed with the bait gene and 1 ml of *B. pervariabilis × D. grandis* cDNA library (Y187) were combined in a 2 L conical flask. A 45 ml volume of 2 × YPDA was added, and the mixture was incubated at 30°C and 40 r/min for 24 h. The bacteria were centrifuged at 1,000 × *g* for 10 min in a 50 ml centrifuge tube, collected, and resuspended in 10 ml of 0.5× YPDA solution (Kana concentration at 50 μg/ml). Next, the bacteria were coated with SD/−Trp/−Leu/X-α-Gal/AbA (DDO/X/A) and SD/−Trp/−Leu/-His/X-α-Gal/AbA (TDO/X/A) separately and cultured on/AbA (TDO/X/A)-deficient medium. After approximately 3–5 days, the blue yeast monoclones were selected onto SD/−Trp/−Leu/-His/−Ade/X-α-Gal/AbA (QDO/X/A) plates for further screening. Positive clones growing on QDO/X/A were sequenced and verified one-to-one.

##### Validation of one-to-one interaction between bait plasmid and prey plasmid

Plasmid DNA was extracted from the blue colonies growing on the QDO/X/A medium using the yeast plasmid DNA extraction kit. The extracted plasmid DNA was transferred into *E. coli* DH5α receptor cells and coated with ampicillin medium to screen the bacteria transferred into pGADT7. The plasmid sequences were compared with data from the NCBI database, and the gene information represented by the plasmid sequences was clarified. The clones most likely related to the function of *ApCtf1β* were selected and validated based on the gene information. To exclude false positives and obtain proteins with definite interactions with *ApCtf1β*, candidate proteins screened from the library were constructed on the pGADT7 vector and then co-transferred into yeast cells coated with QDO/X/A medium and with the decoy plasmid. We observed whether and to what extent the yeast colonies turned blue to verify the interaction further.

#### BiFC verification of protein interactions

##### Gene cloning and vector construction

Specific primers were designed based on the sequences of *BDUbc*, *BDNADP-ME*, *BDWGA-3*, *BDSKL1*, and *BDGolS2* genes in the NCBI database (SAMN19312317; Supplementary Table S1). PCR amplification was performed using *B. pervariabilis × D. grandis* cDNA as a template. The PCR products were detected *via* 1% agarose gel electrophoresis, and the PCR products were recovered and ligated to the cloning vector pEASY-Blunt Zero to obtain the recombinant plasmids. The ligated products were transformed into receptor cells DH5α. The positive transformants were picked after correct verification *via* colony PCR and sent to Tsingke. We designed pSPYNE (R)173-*ApCtf1β*, pSPYCE (M)-*BDUbc*, pSPYCE (M)-*BDNADP-ME*, pSPYCE (M)-*BDWGA-3*, pSPYCE (M)-*BDSKL1*, and pSPYCE (M)-*BDGolS2* homologous recombination primers (Supplementary Table S1). PCR reactions were performed using the cloned plasmids successfully sequenced in the previous step as templates, and the PCR products were recovered. The pSPYNE (R)173 and pSPYCE (M) vectors were recovered following overnight digestion with the restriction endonuclease BamHI at 37°C. The recovered *ApCtf1β* fragment was ligated with linearized pSPYNE (R)173 using homologous recombinase. *BDUbc*, *BDNADP-ME*, *BDWGA-3*, *BDSKL1*, and *BDGolS2* fragments were ligated with the linearized pSPYCE (M) vector at 50°C for 15 min, transferred into *E. coli* receptor cells DH5α, and subjected to colony PCR for identification. The plasmids were extracted from the positive clones and sent to Tsingke for sequencing. The sequences were compared with the correct ones to confirm that the target genes were not mutated.

##### BiFC validation of *ApCtf1β* and candidate interaction genes in *Nicotiana benthamiana*

*Nicotiana benthamiana* plants grown at 25°C and incubated for approximately 30 days were selected for *Agrobacterium* infiltration experiments. The constructed BiFC recombinant vector was transformed into *Agrobacterium tumefaciens* GV3101 (pSoup-19) using the freeze–thaw method (Weigel and Glazebrook, 2006). The monoclonal *Agrobacterium* containing the target vector was picked into 5 ml of LB medium containing the corresponding antibiotics and cultured until the logarithmic phase of *Agrobacterium* growth was reached (OD_600_ = 0.5–0.6). Bacteria were collected following centrifugation at 5,000 rpm for 10 min at 25°C, resuspended in an immersion solution (containing 10 mM MgCl_2_, 10 mM MES, and 150 μM acetosyringone; pH = 5.6) to an OD_600_ of 1.0, and left to rest at room temperature for 2–3 h. Equal volumes of two bacteriophages containing different plasmids were mixed. A small opening was made on the back of a *N. benthamiana* leaf using a 1-mm needle. The bacteriophage was aspirated using the needle and injected into the leaf at the wound site. The water-stained areas of the tobacco leaves were marked. After the infiltrated plants were incubated at approximately 21°C for 2 days, observations were made under laser confocal microscope (FV3000, Olympus, Tokyo, Japan), 488 nM excitation and 509 nM emission filters were used. The genes bZIP63 and bZIP1, which have been shown to interact under laboratory conditions, were used as positive controls. The experimental setup is shown in Table 2.

**Table 2:** BiFC experimental setup.

Plasmid	Experimental setup												
pSPYNE(R)173-bZIP63	+	−	−	−	−	−	−	−	−	−	−	−	−
pSPYCE(M)-bZIP1	+	−	−	−	−	−	−	−	−	−	−	−	−
pSPYNE(R)173-*ApCtf1β*	−	+	+	+	+	+	+	−	−	−	−	−	−
pSPYCE(M)-*BDUbc*	−	+	−	−	−	−	−	+	−	−	−	−	−
pSPYCE(M)-*BDNADP-ME*	−	−	+	−	−	−	−	−	+	−	−	−	−
pSPYCE(M)-*BDWGA-3*	−	−	−	+	−	−	−	−	−	+	−	−	−
pSPYCE(M)-*BDSKL1*	−	−	−	−	+	−	−	−	−	−	+	−	−
pSPYCE(M)-*BDGolS2*	−	−	−	−	−	+	−	−	−	−	−	+	−
pSPYNE(R)173	−	−	−	−	−	−	−	+	+	+	+	+	+
pSPYCE(M)	−	−	−	−	−	−	+	−	−	−	−	−	+

+, means the plasmid is in the system; −, means the plasmid is not in the system.

#### GST pull-down assay to validate protein interactions

##### Gene cloning and vector construction

The homologous recombinant primers for PGEX-6P-1-*ApCtf1β*, pET28a-*BDUbc*, and pET28a-*BDSKL1* were designed (Supplementary Table S1), and the PCR reaction was performed using the cloned plasmids successfully sequenced in the previous step as templates. The PGEX-6P-1 and pET28a vectors were recovered following overnight digestion with the restriction endonuclease BamHI at 37°C. The recovered *ApCtf1β* fragment was ligated with the linearized PGEX-6P-1 vector, while *BDUbc* and *BDSKL1* fragments were ligated with the linearized vector pET28a at 50°C for 15 min. The plasmids were transferred into *E. coli* receptor DH5α for colony PCR identification. The positive clones were extracted from the plasmids, sent to Tsingke for sequencing, and compared with the correct sequences to confirm that no mutations were found in the target gene.

##### Expression and purification of fusion proteins

Primary nuclear expression and purification of GST-*ApCtf1β*, His-*BDUbc*, and His-*BDSKL1* fusion proteins were performed according to the method described by Xu (2020). The eluate containing purified proteins was concentrated and dialyzed, and 10-μl samples were subjected to SDS-PAGE.

##### GST pull-down assay

The GST pull-down experiment was performed according to the method described by Xu. The Glutathione-agarose resin was first treated with GST-tagged protein mixed with glutathione-agarose resin homogenate for the control group and GST-*ApCtf1β* fusion protein mixed with glutathione-agarose resin homogenate for the experimental group. Next, His-*BDUbc* or His-*BDSKL1* fusion proteins were added to the control and experimental groups, respectively. The supernatants were collected following overnight incubation with agitation. Finally, the supernatant was separated by SDS-PAGE, and the protein bands were transferred onto PVDF membranes *via* the wet transfer method for immunoblotting.

#### Bioinformatics analysis of *BDUBC* and *BDSKL1*

Blast^1^ was used for sequence homology comparisons, and ExPASy’s ProtParam tool^2^ was used for the physicochemical property (isoelectric point, molecular weight) analysis of proteins, Predictprotein^3^ was used for secondary structure prediction of the protein encoded by the gene, and SWISS-MODEL^4^ was used for tertiary structure prediction of the protein encoded by this gene. MEGA 7 was used for amino acid sequence homology and phylogenetic analysis, and a neighbor-joining evolutionary tree was constructed with 1,000 replicates and under all other default settings.

#### Expression analysis of *BDUbc* and *BDSKL1* using qRT-PCR after *Arthrinium phaeospermum* infestation

*Bambusa pervariabilis × D. grandis* shoots inoculated with *A. phaeospermum* for 0, 8, 24, 72, 120, 168, and 240 h were used as starting material. RNA was extracted and reverse transcribed to cDNA. Real-time fluorescent quantitative primers q*BDUbc*-F/R and q*BDSKL1*-F/R were designed with reference to the *BDUbc* and *BDSKL1* sequences (Supplementary Table S1). Three software programs, geNorm, Normfinder, and BestKeeper, were used to statistically analyze the expression stability of candidate reference genes (Li et al., 2021) in the *B. pervariabilis × D. grandis* after infestation by *A. phaeospermum* (Primers were shown in Supplementary Table S1; Vandesompele et al., 2002; Silver et al., 2006; Xie et al., 2012). To determine the optimal number of reference genes, the GeNorm program was used to determine whether increasing the number of reference genes could improve the stability of the normalization factor by calculating the pairwise variation values V_n/Vn + 1_ of different candidate genes using the rank order normalization factors NF_n_ and NF_n + 1_ based on the stability of different gene expression (Vandesompele et al., 2002). TransScript Green One-Step qRT-PCR SuperMix (TransGen) was used for qRT-PCR, with three reactions for each treatment group. The mean values were calculated, and the data were analyzed using the 2^-ΔΔCt^ method.

## Results

### Yeast library recombination rate and insert length identification

**Figure 1:**
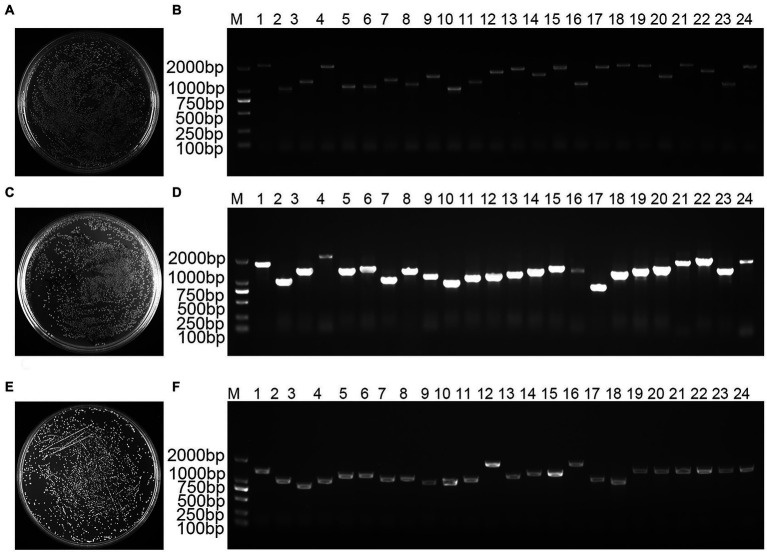
*Bambusa pervariabilis × Dendrocalamopsis grandi* cDNA library quality assay. **(A)** Primary library titer assay at 100-fold dilution; **(B)** PCR detection of primary library insert cDNA fragments, 1–24: 24 randomly selected single clones from the primary library; **(C)** secondary library titer assay at 100-fold dilution; **(D)** PCR for secondary library insert cDNA fragment, 1–24: 24 randomly selected monoclonal clones from secondary library; **(E)** Yeast working solution library titer assay at 100-fold dilution; and **(F)** PCR for yeast working solution insert library fragment, 1–24: 24 randomly selected monoclonal clones from yeast working solution; M: DL2000 DNA Marker.

mRNA analysis showed a homogeneous diffuse elastic distribution of the bands in the range of 500–2,000 bp, demonstrating that the quality of the mRNA bands of the validated cDNA library was well maintained without chemical degradation (Figure 1). Double-stranded (ds) cDNA bands showed a diffuse homogeneous longitudinal distribution of the structure over a wide length range, indicating that the structural integrity of the cDNA bands was satisfactory and the quality performance was high (Supplementary Figure S1). A total of 1,400 monoclonal clones were counted on medium, and the total library capacity was 1.12 × 10^7^ CFU (Figure 1A). The 24 monoclonal clones randomly selected for colony PCR were successfully amplified, and the average length of the amplified fragments was greater than 1,000 bp, indicating that the cDNA insertion rate in the primary library was 100% (Figure 1B). A total of 1,500 monoclonal clones were counted on medium, the secondary library titer was calculated to be 3.00 × 10^6^ cfu/ml (Figure 1C), and the total library capacity was 1.20 × 10^7^ CFU. The 24 monoclonal clones randomly selected for colony PCR were successfully amplified, and the average length of the amplified fragments was greater than 1,000 bp, indicating that the insertion rate of cDNA in the secondary library was 100% (Figure 1D). The results showed that the constructed library capacity was large enough to meet the library requirements for screening the reciprocal proteins. A total of 800 monoclones were counted on the medium showed that the yeast working solution titer was 8.00 × 10^7^ cfu/ml (Figure 1E). The 24 monoclones randomly selected for colony PCR were successfully amplified with a 100% positive rate (Figure 1F). This yeast library can be used for downstream yeast two-hybrid screening.

### Identification of bait plasmids

The *ApCtf1β* gene sequence was amplified from the *A. phaeospermum* cDNA and was 1,236 bp in length. pGBKT7-*ApCtf1β* was obtained by homologous recombination of the *ApCtf1β* fragment with pGBKT7. The plasmid was sequenced, verified positive and without base mutations (Supplementary Figure S2).

### Bait protein toxicity and self-activation assays

The result showed that the positive control strain grew on the QDO/X/A medium and was blue, indicating that it could activate the AbA and X-α-Gal reporter genes; therefore, the positive control experiment was successful (Supplementary Figures S3 A,B). The negative control strain grew well on the DDO medium but failed to grow on the QDO/X/A medium, indicating that it could not activate the AbA and X-α-Gal reporter genes; therefore, the negative control experiment was successful (Supplementary Figures S3 C,D). The decoy pGBKT7-*ApCtf1β* and pGADT7 grew on DDO/X medium and were blue, indicating that the pGBKT7-*ApCtf1β* plasmid was successfully transferred into the yeast strain. The normal size of the transformed yeast cells indicated that the decoy plasmid was not toxic. pGBKT7-*ApCtf1β* grew blue on TDO/X but not on QDO/X/A, indicating that pGBKT7-ApCtf1β had slight self-activation but can be inhibited under QDO/X/A screening conditions. It can thus be used for subsequent screening experiments that include QDO/X/A screening (Supplementary Figures S3 E–G).

### Two-hybrid library screening using yeast mating

The results showed that yeast broth produced approximately 144 positive clones on the QDO/X/A medium (Supplementary Figures S4 A,B). The blue clones on the QDO/X/A screening plate were picked and transferred to new QDO/X/A screening plates for further screening. A total of 122 clones were obtained growing blue (Supplementary Figures S4 C,D), and PCR products were sequenced. All sequencing results are shown in Supplementary Table S2. The sequencing results were combined to exclude duplicates, shifts, and clones with non-coding regions. Seven significant sequences were screened: *BDUbc* (ubiquitin-conjugating enzyme), *BDNADP-ME*(NADP-dependent malic enzyme), *BDWGA-3*(agglutinin isolectin 3), *BDSKL1*(shikimate kinase-like protein 1), *BDBBTI*(Bowman-Birk type bran trypsin inhibitor-like), *BDnsLTP1*(non-specific lipid transfer protein-like 1), and *BDGolS2*(galactinol synthase 2).

### Yeast two-hybrid validated protein interactions

**Figure 2:**
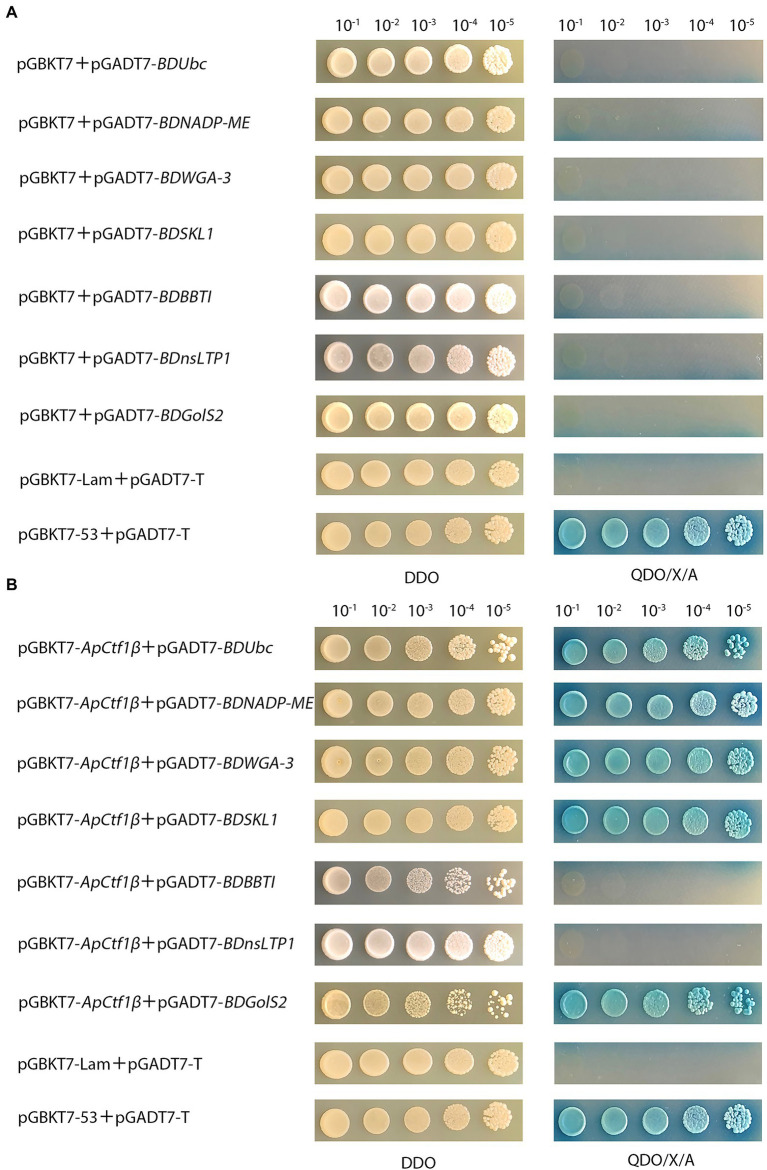
**(A)** Self-activation validation of positive candidate gene from gradient dilution spot medium; pGBKT7 + pGADT7-*BDUbc*, pGBKT7 + pGADT7-*BDNADP-ME*, pGBKT7 + pGADT7-*BDWGA-3*,pGBKT7 + pGADT7-*BDSKL1*,pGBKT7 + pGADT7-*BDBBTI*, pGBKT7 + pGADT7-*BDnsLTP1*, and pGBKT7 + pGADT7-*BDGolS2* were the experimental groups; pGBKT7-53 + pGADT7-T was the positive control; pGBKT7-Lam + pGADT7-T was the negeative control; **(B)** Validation of positive candidate gene from gradient dilution spot medium. pGBKT7-*ApCtf1β* + pGADT7-*BDUbc*, pGBKT7-*ApCtf1β* + pGADT7-*BDNADP-ME*, pGBKT7-*ApCtf1β* + pGADT7-*BDWGA-3*, pGBKT7-*ApCtf1β* + pGADT7-*BDSKL1*, pGBKT7-*ApCtf1β* + pGADT7-*BDBBTI*, pGBKT7-*ApCtf1β* + pGADT7-*BDnsLTP1*, and pGBKT7-*ApCtf1β* + pGADT7-*BDGolS2* were the experimental groups; pGBKT7-53 + pGADT7-T was the positive control; pGBKT7-Lam + pGADT7-T was the negeative control.

The results showed that yeast strains grew normally on the DDO medium but not on the QDO/X/A medium, indicating that none of these genes were self-activated (Figure 2A).The positive control can grow normally and turn blue on DDO/X/A, while the negative control cannot grow on DDO/X/A, indicating the success of the control experiment. pGBKT7-*ApCtf1β* + pGADT7-*BDUbc*, pGBKT7-*ApCtf1β* + pGADT7-*BDNADP-ME*, pGBKT7-*ApCtf1β* + pGADT7-*BDWGA-3*, pGBKT7-*ApCtf1β* + pGADT7-*BDSKL1*, and pGBKT7-*ApCtf1β* + pGADT7-*BDGolS2* could grow blue clones on QDO/X/A medium. pGBKT7-*ApCtf1β* + pGADT7-*BDBBTI* and pGBKT7-*ApCtf1β* + pGADT7-*BDnsLTP1* did not grow blue clones when transformed into QDO/X/A medium, proving that the reporter gene was not activated (Figure 2B). Therefore, *ApCtf1β* can interact with *BDUbc*, *BDNADP-ME*, *BDWGA-3*, *BDSKL1*, and *BDGolS2*; however, there is no interaction with *BDBBTI* and *BDnsLTP1*.

### BiFC assay verifies protein interactions *in vivo*

**Figure 3:**
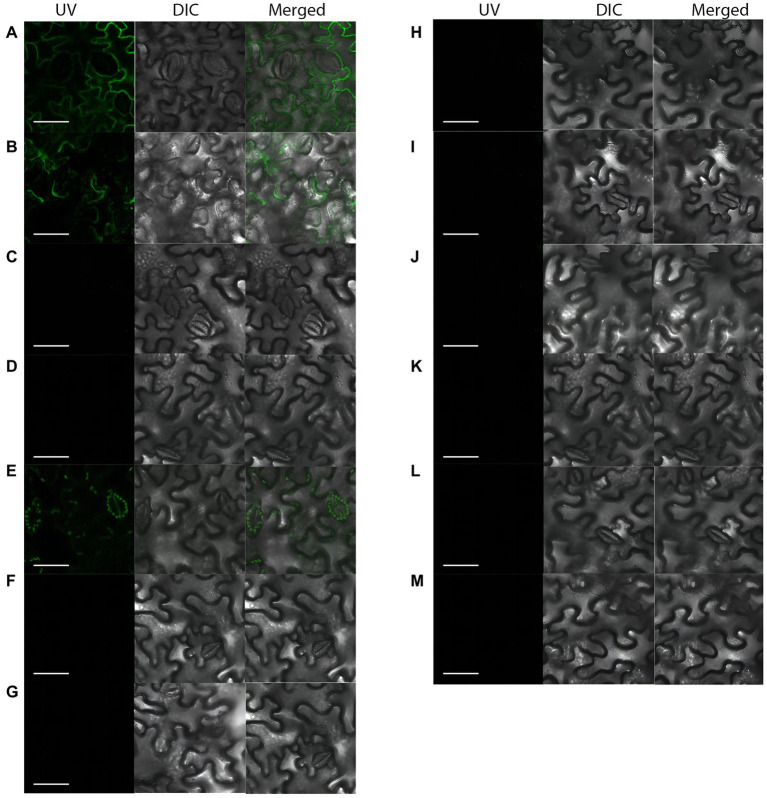
Fluorescent laser confocal detection of *Nicotiana benthamiana* epidermal cells. **(A)** pSPYNE(R)173-*bZIP63 +* pSPYCE(M)-*bZIP1*; **(B)** pSPYNE(R)173-*ApCtf1β +* pSPYCE(M)-*BDUbc*; **(C)** pSPYNE(R)173-*ApCtf1β +* pSPYCE(M)-*BDNADP-ME*; **(D)** pSPYNE(R)173-*ApCtf1β +* pSPYCE(M)-*BDWGA-3*; **(E)** pSPYNE(R)173-*ApCtf1β +* pSPYCE(M)-*BDSKL1*; **(F)** pSPYNE(R)173-*ApCtf1β +* pSPYCE(M)-*BDGolS2*; **(G)** pSPYNE(R)173 *+* pSPYCE(M); **(H)** pSPYNE(R)173-*ApCtf1β +* pSPYCE(M); **(I)** pSPYNE(R)173 + pSPYCE(M)-*BDUbc*; **(J)** pSPYNE(R)173 + pSPYCE(M)-*BDNADP-ME*; **(K)** pSPYNE(R)173 + pSPYCE(M)-*BDWGA-3*; **(L)** pSPYNE(R)173 + pSPYCE(M)*BDSKL1*; **(M)** pSPYNE(R)173 + pSPYCE(M)-*BDGolS2*; **(A)** positive control; **(B–F)** experimental groups; **(G–M)** negative controls; UV, GFP fluorescence; DIC, bright field; and Merged, superimposition of GFP fluorescence and bright field.

The *ApCtf1β* gene sequence was amplified from the *A. phaeospermum* cDNA with a full gene length of 1,236 bp. The *BDUbc*, *BDNADP-ME*, *BDWGA-3*, *BDSKL1*, and *BDGolS2* gene sequences were amplified from *B. pervariabilis* × *D. grandis* cDNA with lengths of 591, 1,713, 624, 573, and 1,008 bp, respectively. PCR of the bacteriophage solution matched the theoretical values, and the sequencing was verified as positive and without base mutations (Supplementary Figure S5).The results showed that a strong fluorescent signal was detected using laser confocal microscopy in the positive control. In contrast, no fluorescent signal was obtained for the negative control, showing the success of the experimental setup. Compared to the negeative control, which had no fluorescent signal in the cells, the transient expressed pSPYNE(R)173-*ApCtf1β* + pSPYCE(M)-*BDUbc* and pSPYNE(R)173-*ApCtf1β* + pSPYCE(M)-*BDSKL1* showed a strong fluorescent signal in *N. benthamiana* cells (Figure 3). These findings demonstrate that the decoy protein *ApCtf1β* interacts with the prey proteins *BDUbc* and *BDSKL1* in *N. benthamiana* cells in the stomata but not with the three proteins *BDNADP-ME*, *BDWGA-3*, and *BDGolS2*. Therefore, two prey proteins, *BDUbc* and *BDSKL1*, were selected for *in vitro* validation.

### GST pull-down assay validates protein interactions

**Figure 4:**
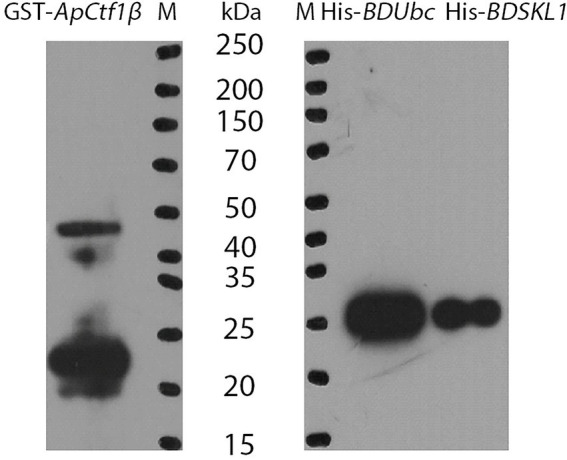
Immunoblot analysis of fusion proteins. M: Protein Marker.

**Figure 5:**
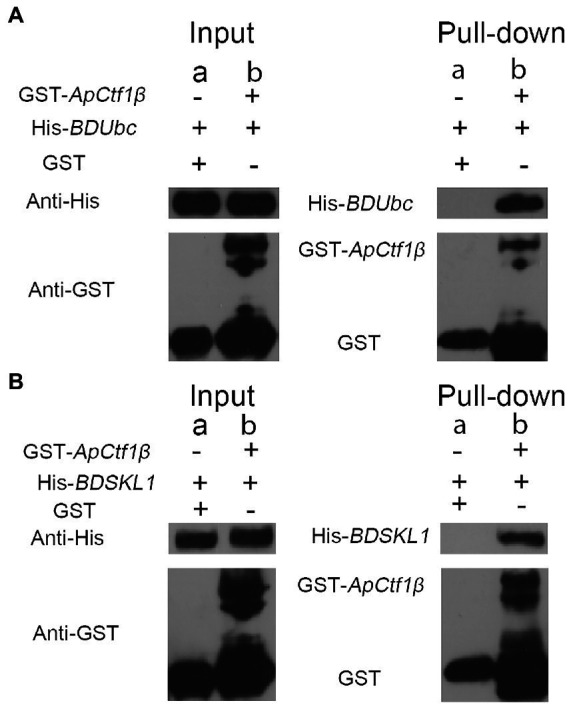
**(A)** GST pull-down assay of *ApCtf1β* interacting with the *BDUbc* protein *in vitro*; **(B)** GST pull-down assay of *ApCtf1β* interacting with the *BDSKL1* protein *in vitro*; a: control group; b: experimental group.

The *ApCtf1β* gene sequence amplified from the cDNA of *A. phaeospermum* was 1,236 bp long. Amino acid sequence sorting showed that *ApCtf1β* had no signal peptide or transmembrane region and that there was a long continuous disordered sequence at the C-terminus. Therefore, *ApCtf1β* was intercepted at 1–217 aa for expression, i.e., 651 bp. The *BDUbc* and *BDSKL1* sequences were amplified from the *B. pervariabilis* × *D. grandis* cDNA, producing fragments 591 and 573 bp long, respectively. Following amino acid sequence sorting, *BDUbc* and *BDSKL1* had no signal peptide, transmembrane region, and low disorder. Therefore, they were expressed at full length, i.e., 197 and 191 aa, respectively. PCR using both bacterial broths produced fragments between 500 and 750 bp in length, consistent with theoretical values, and were sequenced and verified as positive without base mutations (Supplementary Figure S6). The GST-*ApCtf1β* fusion protein was expected to be 49 kDa in size, as expected by SDS-PAGE (Figure 4). The His-*BDUbc* and His-*BDSKL1* fusion proteins were expected to be 21.31 and 20.89 kDa in size, respectively. The actual SDS-PAGE results showed a target band of the expected size (Figure 4). This result indicates that the GST-*ApCtf1β*, His-*BDUbc*, and His-*BDSKL1* fusion proteins were successfully obtained and that the GST-pull down experiment can be used in further investigations. The results of the *ApCtf1β* and *BDUbc* protein *in vitro* interaction experiment are shown in Figure 5A. The hybridization results of the corresponding His-tagged antibody showed that the eluted control group did not contain the His-*BDUbc* fusion protein, indicating that the *BDUbc* protein and the GST-tagged protein did not interact and, therefore, could not be adsorbed on the GST column. The experimental group contained the His-*BDUbc* fusion protein, indicating that the *BDUbc* protein and GST-*ApCtf1β* fusion protein in *ApCtf1β* interactively bound to adsorb on the GST column. Similarly, the results of the *in vitro* interaction assay between the *ApCtf1β* and *BDSKL1* proteins are shown in Figure 5B. The hybridization results of the his-tagged antibody showed that the eluted control group did not contain the His-*BDSKL1* fusion protein and that the experimental group contained the His-*BDSKL1* fusion protein, thus indicating that *BDSKL1* protein can interact with *ApCtf1β* in GST-*ApCtf1β* fusion protein and adsorb to the GST column. The GST pull-down assay results showed that the *ApCtf1β* protein can interact with the *BDUbc* and *BDSKL1* proteins *in vitro*.

### Bioinformatics analysis of *BDUbc* and *BDSKL1*

**Figure 6:**
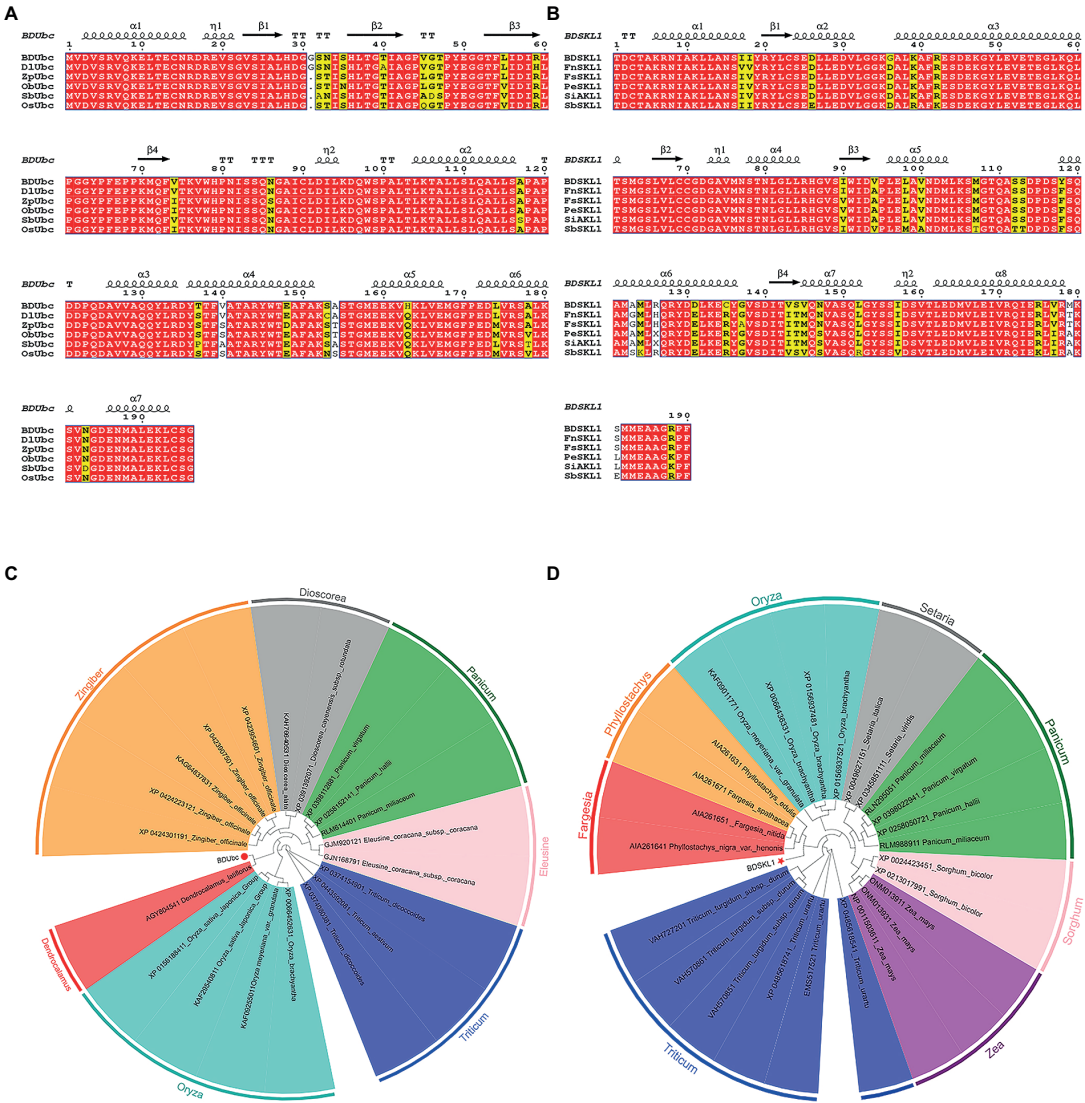
Multiple alignment of proteins and target gene phylogenetic tree based on neighbor-joining algorithm. **(A)** Multiple alignment of the obtained *BDUbc* protein with *BDUbc* proteins from other plants; **(B)** Multiple alignment of the obtained *BDSKL1* protein with *BDSKL1* proteins from other plants. Completely identical amino acid sequences are indicated using white words and red backgrounds; highly conserved amino acid sequences are represented by black words and yellow backgrounds; non-conserved amino acid sequences are represented by gray words and white backgrounds. α-Helices are displayed as large squiggles and η-helices as small squiggles; strict β-turns are shown as TT letters and β-strands as arrows. Strictly conserved amino acid residues are shown as. **(C)** *BDUbc* phylogenetic tree; **(D)** *BDSKL1* phylogenetic tree. The numbers above/below the nodes indicated bootstrap number.

**Figure 7:**
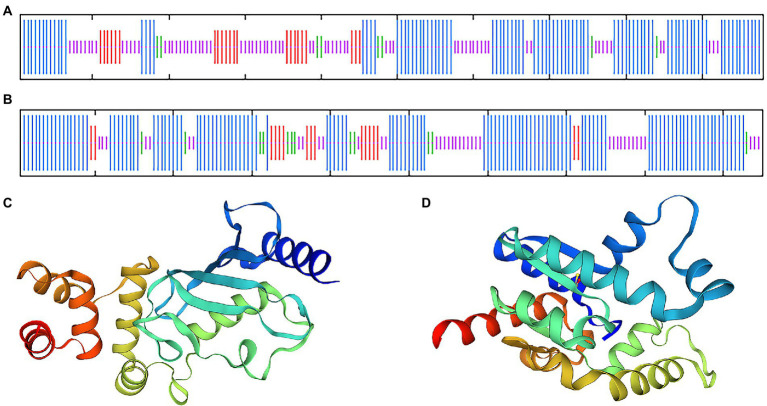
Transmembrane region, signal peptide, hydrophobicity, structure prediction of *BDUbc* and *BDSKL1*. **(A,B)** Secondary structure prediction of *BDUbc* and *BDSKL1,* α-helix, random coil, extended strand, and β-turn are represented with blue, pink, red and green regions; **(C,D)** tertiary structure prediction of *BDUbc* and *BDSKL1*.

Blast and multiple alignment analysis using ClustalW revealed that *BDUbc* and *BDSKL1* have a high identity with other reported plant Ubcs and SKL1s in GenBank. *BDUbc* shares 97.45% identity with *DlUbc* (AGY80454.1), 93.88% identity with *ZpUbc*(KAG8054101.1), *ObUbc*(XP006645263.1), *SbUbc* (XP002459014.1), and *OsUbc* (XP015618841.1), respectively (Figure 6A). *BDSKL1* shares 93.16, 92.63, 91.58, 88.42, and 88.59% identity with *FnSKL1* (AIA26165.1), *FsSKL1* from (AIA26167.1), *PeSKL1* from (AIA26163.1), *SiSKL1* from *Setaria italica* (AIA26163.1), and *SbSKL1* from (XP_002442345.1), respectively (Figure 6B). The results from the database were compared using the NCBI online blast nucleic acid sequence analysis tool. Comparison analysis using MEGA 5.1 showed that the annotation of different families, genera, and model plants was selected as the phase protein gene sequence. The phylogenetic tree was constructed using the neighbor-joining algorithm, and the results showed that *BDUbc* was more closely related to *Dendrocalamus latiflorus*, while *BDSKL1* was more closely related to the bamboo subfamily (Figures 6C,D). The basic physicochemical properties of the proteins encoded by the *BDUbc* and *BDSKL1* genes were analyzed using ProtParam, The numbers of amino acid residues of *BDUbc* and *BDSKL1* were 196 and 190, the instability coefficients were 41.68 and 34.93, the theoretical isoelectric points were 5.06 and 4.72, and the predicted molecular weights were 21.31 and 20.89 kDa, respectively. The amino acid sequences were analyzed for hydrophilicity using Protscale, and both proteins were presumed to be hydrophilic. Signal peptide and transmembrane domain prediction using online software SignalP-5.0 and TMHMM showed that both proteins have no signal peptide and no transmembrane structural domain. The secondary structure prediction of the amino acid sequence encoded by *BDUbc* and *BDSKL1* through the SPOMA website showed that the *BDUbc* protein contained α-helix (47.96%), extended strand (11.22%), and random coil (36.73%). *BDSKL1* contained α-helix (63.68%), extended strand (8.68%), extended strand (8.42%), β-turn (6.32%), and random coil (21.58%; Figures 7A,B). SWISS-MODEL was used to model the tertiary structural homology of the translated proteins of *BDUbc* and *BDSKL1* (Figures 7C,D). The comparison results showed that the *BDUbc* global model quality assessment (GMQE) score was 0.67, and QMEANDisCo global score was 0.69 ± 0.06. The *BDSKL1* global model quality assessment (GMQE) score was 0.80, and QMEANDisCo global score was 0.78 ± 0.06. Initially, the functions were determined to have similarities.

### qRT-PCR validation of *BDUbc* and *BDSKL1* gene expression in *Bambusa pervariabilis* × *Dendrocalamopsis grandis* after infestation with *Arthrinium phaeospermum*

**Figure 8:**
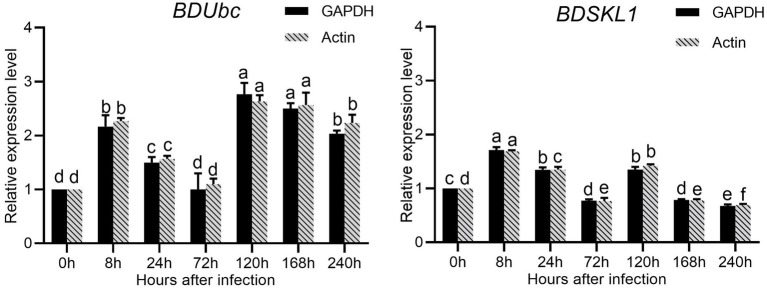
Relative expression levels of *BDUbc* and *BDSKL1*.There are significant differences in the relative expression of different lowercase letters (*p* < 0.05).

The results were shown in the Supplementary Table S3 calculated that both *GAPDH* and *Actin* were the two most stable expression genes. The pairwise variation values of all candidate reference genes were less than the threshold value of 0.15, and the value of V_2/3_ was 0.045, which was smaller than 0.15, indicating that two reference genes were sufficient for accurate normalization of these samples sufficient (Supplementary Figure S8). Therefore, we chosed *GAPDH* and *Actin* as double reference to analyze the changes in expression. The results showed that the expression of *BDUbc* increased significantly at 8 h after infestation, then decreased slowly, with the highest expression at 72 h. The relative expression at 240 h was approximately twice as high as that at 0 h. The expression of *BDSKL1* increased significantly at 8 h after infestation to reach the highest value, reached the lowest at 72 h, increased significantly at 120 h, and then decreased slowly. The relative expression at 240 h was lower than at 0 h (Figure 8).

## Discussion

High-quality cDNA libraries are a strong guarantee for screening reciprocal proteins in yeast two-hybrid systems, and cDNA library quality depends mainly on RNA integrity and library capacity (Xu et al., 2021). Plants often exhibit high expression of disease-resistance-associated genes when infested with pathogens. These genes are expressed in relatively low abundance without pathogen stimulation and are potential targets for disease-causing genes in response to pathogen infestation. Therefore, yeast libraries were selected from bamboo hybrid shoots infested with *A. phaeospermum* spore suspension for 168 h to provide a more comprehensive picture of the expression of genes in yeast libraries. A study reported that the library capacity of the yeast two-hybrid library constructed using *Phyllostachys pupescens* was 1.2 × 10^6^ CFU with a 99% recombination rate (Zheng and Ming-bing, 2012), while the library capacity of *Dendrocalamus sinicus* was 1.04 × 10^5^ CFU with a 95% recombination rate (Wang et al., 2006). The average length of both libraries was approximately 1,000 bp. By contrast, the library capacity of the *B. pervariabilis × D. grandi* yeast secondary library was 1.20 × 10^7^ CFU, with inserts averaging over 1,000 bp and a recombinant insertion rate of 100%, all of which were slightly higher than those of other bamboo subfamilies, and could satisfy the screening of intercalated proteins with low expression in abundance. The CDS sequence analysis of the two intercalated proteins showed that both were full-length CDS insertions into the secondary library. The above results indicate that the constructed *B. pervariabilis × D. grandi* library is of good quality and can be used to screen host target proteins for the keratinase transcription factor *ApCtf1β* of *A. phaeospermum* and to provide a database for the subsequent screening of the reciprocal target proteins of other related pathogenic genes of *A. phaeospermum* in *B. pervariabilis × D. grandi* and their mechanism.

*Bambusa pervariabilis × D. grandi* has a well-developed cuticle, which, in addition to its barrier function of water retention and cleansing, has a complex defense function of promoting overall plant development and regulating plant-pathogen interactions (Dickman, 1986; Barthlott and Neinhuis, 1997; Serrano et al., 2014). *Arthrinium phaeospermum* must degrade the cuticle to invade *B. pervariabilis × D. grandi* by secreting cuticle-degrading enzymes, including esterases, cutinases, and lipases, which catalyze the hydrolysis of ester bonds in lipoproteins, fats, and waxes to penetrate the outermost cuticle barrier. Cutinases are considered important enzymes, as they allow the fungus to penetrate the cuticle and infect the plant and function as an attachment to the plant epidermis, cuticle invasion, and signal production (Beys da silva et al., 2010; Ortiz-Urquiza and Keyhani, 2013). The cutinase transcription factor is a gene that was significantly up-regulated when in the growth environment of the host plant culture, as identified by our previous transcriptome sequencing analysis of *A. phaeospermum* under different culture conditions. Furthermore, the gene was found to be a key pathogenic gene for *A. phaeospermum* infestation of *B. pervariabilis × D. grandi* using knockdown and backfill tests. Therefore, *ApCtf1β* was used as a bait to screen for and validate two interacting proteins, *BDUbc*, belonging to the plant ubiquitin/protease pathway, and *BDSKL1*, belonging to the shikimate pathway.

The plant ubiquitin/proteasome system is the main pathway for intracellular protein degradation and plays a vital role in plant growth, development, morphogenesis, and disease resistance (Hershko, 2005). Recent studies have also shown that certain pathogenic microorganisms can mimic components of the host plant’s ubiquitin/protease system, thereby exploiting the system to benefit the pathogen (Dielen et al., 2011). The ubiquitin/proteasome pathway consists mainly of the ubiquitin-activating enzyme (E1), ubiquitin-conjugating enzyme (E2), ubiquitin-protein ligase (E3), proteasomes, and deubiquitinases (DUBs). The reaction process starts with activating the ubiquitin molecule by E1 using ATP. The activated ubiquitin molecule is attached to E1 by a thioester bond. E1 further binds to E2 and transfers the ubiquitin molecule to E2 *via* cross-esterification. E2 then binds to E3, mainly responsible for recruiting the substrate. Thus, the substrate is labeled with ubiquitin and later degraded by the proteasome into small polypeptides. Simultaneously, the ubiquitin molecule is re-cycled *via* the action of a deubiquitinating enzyme (Zeng et al., 2006; Stone, 2014). Ubiquitination plays an essential role in the plants’ biotic and abiotic stress responses (Zeng et al., 2006; Dielen et al., 2011; Marino et al., 2012; Stone, 2014). It has been known as early as 2006 that the ubiquitin/proteasome system may play an important role in plant-microbe interactions (Zeng et al., 2006), and much indirect evidence is available. Next, Dielen et al. (2011), published in *Molecular Phytopathology*, emphasize that the ubiquitin/proteasome system is involved in every step of the plant defense response. The ubiquitin/proteasome system is not only a defense weapon for the host plant but also a target for some pathogens to attack, thereby inhibiting the normal functioning of the system or using it for the benefit of the pathogen. Therefore, it is easy to propose a scenario in which the pathogen disrupts the ubiquitin/proteasome system of the host plant while infesting the plant, thereby disrupting the homeostasis of the host plant cell and facilitating pathogen infestation or a scenario in which the pathogen secretes effector proteins into the host plant cell, mimicking components of the host plant’s ubiquitin/proteasome system, thus exploiting the host’s ubiquitin/proteasome system for the benefit of the pathogen. The proteasome system is used to facilitate the successful infestation of the pathogen. There is experimental evidence that Os*UBC*26 expression in rice is induced by *P. ramorum* and JA treatment. Zhiguo et al. (2015) also showed that the expression of rice ubiquitin-conjugating enzymes could be induced by at least two phytohormones among IAA, 6-BA, GA, and ABA, further suggesting that the rice ubiquitin-conjugating enzyme E2 are involved in response to the infestation signaling pathway of rice blast and are likely to be associated with the JA signaling pathway. The ubiquitin-conjugating enzyme E2 mainly determines the length and topology of the ubiquitin chain, and ubiquitin chain-tagged protein molecules with different topologies will perform different functions. For example, ubiquitin chain-tagged protein molecules linked to K48 are mainly sent to the 26S proteasome for degradation, whereas ubiquitin chain-tagged protein molecules linked to K63 primarily function in signal transduction. Therefore, based on the qRT-PCR results, we can speculate that ubiquitin-binding enzyme genes induced at the early stage of *A. phaeospermum* infestation are likely to play a role in signaling the disease resistance defense response in *B. pervariabilis × D. grandi*. Conversely, ubiquitin-conjugating enzyme genes that were induced later are likely to play a role in initiating the defense response in *B. pervariabilis × D. grandi*, for example, by degrading certain defense-response-related repressor proteins through the ubiquitination pathway, thereby initiating the *B. pervariabilis × D. grandi* defense response. Conversely, the ubiquitin-conjugating enzyme genes may play a role in initiating defense responses in bamboo. For example, the ubiquitination pathway degrades several defense response-related repressor proteins, thereby initiating the disease-resistant defense response in *B. pervariabilis × D. grandi*.

The shikimate pathway functions at the critical interface between primary and secondary metabolism by directing carbon from the glycolytic and pentose phosphate pathways to synthesize a wide range of physiologically important aromatic compounds (Le Maréchal and Azerad, 1976). In plants, these include aromatic amino acids, phenylpropanoids, lignans, hormones, pigments, plant antitoxins, alkaloids, UV protectors, and electron carriers (Takeya et al., 1996). The major metabolites of the shikimate pathway are also considered to be blight point substrates for other secondary metabolic pathways. Shikimate kinase (SK) catalyzes the fifth reaction of the manganate pathway, which directs carbon from a central metabolic pool to a wide range of secondary metabolites involved in plant development and growth and stress responses. It is thought that plant SKs act as regulatory points in the mangiferous acid pathway, facilitating metabolic flow to specific secondary metabolite pools (Herrmann, 1995). The rapid induction of plant SK transcripts by fungal inducers (Görlach et al., 1995), significant sensitivity of plant SK activity to cellular ATP energy charge (Collins et al., 2017), and the differential expression of three rice SK genes at specific developmental stages and in response to biological stress have demonstrated that (Kasai et al., 2005). It was shown that *AtSKL1* is a functionally distinct direct homologous cluster evolved from plant SK gene repeats. The *Arabidopsis* genome contains an SKL1 (*AtSKL1*; At3g26900) gene, and the ATP-binding site of *AtSKL1* is highly conserved; however, *AtSKL1* has a structural domain different from the ancestral manganate kinase and lacks the conserved SK manganate binding residues and catalytic residues. Therefore, *AtSKL1* does not catalyze the SK reaction (Fucile et al., 2008). The main role of *AtSK1* is to increase the carbon flux of specific metabolite pools in response to environmental stresses or tissue-specific developmental requirements. For example, tomato SK transcripts are induced approximately 17-fold by fungal excitons, potentially leading to a redirection of carbon flow to plant antitoxin biosynthesis. Moreover, the tomato SK induction pattern is not delayed and is a direct response to infestation with the pathogen (Görlach et al., 1995). Mutations in *AtSKL1* in *Arabidopsis* lead to an albino phenotype, and loss-of-function mutations in SKL1 (e.g., SKL1-8) result in the loss of signals necessary to carry out the nuclear-encoded chloroplast developmental program. Previous studies have shown that a similar down-regulation of photosynthetic gene expression has been reported under different stress conditions (Bilgin et al., 2010). The qRT-PCR results showed that expression of the manganate kinase SKL1 gradually increased after dark spore rhodopsin infestation of *B. pervariabilis × D. grandi*, which may be due to the enhanced photosynthesis of the hybrid bamboo during the pre-invasion period of the pathogen. This occurrence directly induced the biosynthesis of antitoxin substances in pro × green bamboo through the SK mode, followed by a gradual decrease in expression to inhibit the photosynthetic capacity of *B. pervariabilis × D. grandi* in response to plant cells. Moreover, photo-oxidative stress results in induced genes, which are mainly divided into detoxification and stress-related genes, e.g., peroxidases, WRKY, and HSPs. These genes are induced for cellular defense and survival when the plant is under attack by pathogenic microorganisms and under uncomfortable environmental conditions.

In the present study, a cDNA library of *B. pervariabilis × D. grandi* infected by *A. phaeospermum* was constructed for the first time and screened for reciprocal target proteins of *ApCtf1β* in the host using the yeast two-hybrid technique. *In vivo* validation using the BiFC assay and *in vitro* validation using the GST pull-down assay yielded two interaction proteins, *BDUbc* and *BDSKL1*. Our findings have established the foundation for understanding the target protein functions and the resistance mechanism of *B. pervariabilis × D. grandi* in response to pathogenic stress. Moreover, they will provide a reliable theoretical basis to study the pathogenic pathway of *A. phaeospermum* and the molecular mechanism underlying the resistance of hybrid bamboo in response to pathogen infestation. This study has provided a basis for developing new strategies for the sustainable and effective control of forest tree blight wilting diseases.

## Data availability statement

The datasets presented in this study can be found in online repositories. The names of the repository/repositories and accession number(s) can be found at: https://www.ncbi.nlm.nih.gov/genbank/, QYRS0000000.1, MK789640, and MK789641.

## Author contributions

PY and JY: conceptualization. PY: methodology, software, and writing–original draft preparation. SyL, TZ, and SH: validation. PY and XF: formal analysis. TL and JY: investigation. TL: resources. YL and CY: data curation. SjL and TL: writing–review and editing. PY and FH: visualization. SH: supervision. TZ: project administration. SjL: funding acquisition. All authors contributed to the article and approved the submitted version.

## Funding

This research was funded by the National Natural Science Foundation of China, grant number 32171795.

## Conflict of interest

The authors declare that the research was conducted in the absence of any commercial or financial relationships that could be construed as a potential conflict of interest.

## Publisher’s note

All claims expressed in this article are solely those of the authors and do not necessarily represent those of their affiliated organizations, or those of the publisher, the editors and the reviewers. Any product that may be evaluated in this article, or claim that may be made by its manufacturer, is not guaranteed or endorsed by the publisher.

## Footnotes

^1^ http://www.ncbi.nlm.nih.gov/blast/

^2^ http://www.expasy.org/tools/protparam.html

^3^ https://www.predictprotein.org/

^4^ https://www.swissmodel.expasy.org/
