# Supplementary material for: Identification of the interacting proteins of Bambusa pervariabilis × Dendrocalamopsis grandis in response to the transcription factor ApCtf1β in Arthrinium phaeospermum
Source: Front Plant Sci. 2022 Sep 15;13:991077. doi: 10.3389/fpls.2022.991077 (PMC9520005; doi:10.3389/fpls.2022.991077)
Supplement: Supplementary file 1 [file Data_Sheet_1.PDF]

Table S1. Primers used in this study

Primer name	Primer sequences (5'-3')	Sources of references
pGBKT7-*ApCtf1β*-F	TGGAGGCCGAATTCCCGGGATGTGTGTGGCCAAGGCAAAGC	MK789640
pGBKT7-*ApCtf1β*-R	GGTCGACGGATCCCCGGGTTACCCCTTTACCATACCCCCGT	MK789640
BDUbc-F	ATGGTGGACGTGTCGCGCGTGCAG	AGY80454.1
BDUbc-R	TCAGCCGGAGCAGAGCTTTTCAAG	AGY80454.1
BDNADP-ME-F	ATGGCCGGCGGCGGTGTCGAGGACG	ACI46151.1
BDNADP-ME-R	TCACCTGTAGCTGCGGTAGAGCGGGG	ACI46151.1
BDWGA-3-F	ATGATGAAAACCAAGGTCCTCACGC	XP010237391.1
BDWGA-3-R	TTACTCGGTGGTGGAGTTGTTCATG	XP010237391.1
BDSKL1-F	ACGGACTGCACTGCCAAGCGCAACAT	AIA26165.1
BDSKL1-R	CTAGAAGGGCCTTCCAGCAGCTTCCATC	AIA26165.1
BDGolS2-F	ATGGGCCCGAACATGTCGGCGTT	XP004958744.1
BDGolS2-R	CTAGGCTGCCGACGGCGCCGGG	XP004958744.1
pSPYNE(R)173-*ApCtf1β*-F	GGGCCCAGGCCTACTAGTGGATCCATGTGTGTGGCCAAGGCAAAGC	MK789640
pSPYNE(R)173-*ApCtf1β*-R	GGTACCCTCGAGGTCGACGGATCCTTACCCCTTTACCATACCCCCGTC	MK789640
pSPYCE(M)-*BDUbc*-F	GCCTGGCGCGCCACTAGTGGATCCATGGTGGACGTGTCGCGCGTGCAG	AGY80454.1
pSPYCE(M)-*BDUbc*-R	GTCGACAGTACTATCGATGGATCCTCAGCCGGAGCAGAGCTTTTCAAG	AGY80454.1
pSPYCE(M)-*BDNADP-ME*-F	GCCTGGCGCGCCACTAGTGGATCCATGGCCGGCGGCGGTGTCGAGGACG	ACI46151.1
pSPYCE(M)-*BDNADP-ME*-R	GTCGACAGTACTATCGATGGATCCTCACCTGTAGCTGCGGTAGAGCGGGG	ACI46151.1
pSPYCE(M)-*BDWGA-3*-F	GCCTGGCGCGCCACTAGTGGATCCATGATGAAAACCAAGGTCCTCACGC	XP010237391.1
pSPYCE(M)-*BDWGA-3*-R	GTCGACAGTACTATCGATGGATCCTTACTCGGTGGTGGAGTTGTTCATG	XP010237391.1
pSPYCE(M)-*BDSKL1*-F	GCCTGGCGCGCCACTAGTGGATCCACGGACTGCACTGCCAAGCGCAACAT	AIA26165.1
pSPYCE(M)-*BDSKL1*-R	GTCGACAGTACTATCGATGGATCCCTAGAAGGGCCTTCCAGCAGCTTCCATC	AIA26165.1
pSPYCE(M)-*BDGolS2*-F	GCCTGGCGCGCCACTAGTGGATCCATGGGCCCGAACATGTCGGCGTT	XP004958744.1
pSPYCE(M)-*BDGolS2*-R	GTCGACAGTACTATCGATGGATCCCTAGGCTGCCGACGGCGCCGGG	XP004958744.1

PGEX-6P-1-*ApCtf1β*-F	CAGGGGCCCCTGGGATCCATGTGTGTGGCCAAGGCAAAGC	MK789640
PGEX-6P-1-*ApCtf1β*-R	CGGGAATTCCGGGGATCCTTACCCCTTTACCATACCCCCGT	MK789640
pET28a-*BDUbc*-F	CAAATGGGTCGCGGATCCATGGTGGACGTGTCGCGCGTGCAG	AGY80454.1
pET28a-*BDUbc*-R	GAGCTCGAATTCGGATCCTCAGCCGGAGCAGAGCTTTTCAAG	AGY80454.1
pET28a-*BDSKL1*-F	CAAATGGGTCGCGGATCCACGGACTGCACTGCCAAGCGCAACAT	AIA26165.1
pET28a-*BDSKL1*-R	GAGCTCGAATTCGGATCCCTAGAAGGGCCTTCCAGCAGCTTCCATC	AIA26165.1
q*BDUbc*-F	CTTGAAGACGGCGCTGCTAT	AGY80454.1
q*BDUbc*-R	GCACCTTTTCTTCCATGCCA	AGY80454.1
q*BDSKL1*-F	ATGTTCTTGGAGGCAAGGGT	AIA26165.1
q*BDSKL1*-R	TGAGTTCATGACGGCACCAT	AIA26165.1
GAPDH-F	GATTTTGTCGGTGATTCAAGGTC	Li et al., 2021
GAPDH-R	ATGTGAGCGATCAGATCCAGAA	Li et al., 2021
Actin-F	TGACAATGGCACTGGAATGG	Li et al., 2021
Actin-R	CCCATCCCTACCATGACACC	Li et al., 2021
EF1α-F	CGCTGAGATGAACAAGAGGTCG	Li et al., 2021
EF1α-R	CGGTGGTGGAGTCAATGATGAG	Li et al., 2021
PP2A-F	GGGATTTCACCAAGAGGAGC	Li et al., 2021
PP2A-R	CGCTGGATCAAACTGGAGGA	Li et al., 2021
RPL3-F	GAAAGCCTGCCACCTCACTG	Li et al., 2021
RPL3-R	CTTCTCGACCTCACGCACAAT	Li et al., 2021
TIP41-F	GTGCTCTAAAGGCATGGAAACA	Li et al., 2021
TIP41-R	TTCTGAACTTCCATTTCGCTGC	Li et al., 2021

The red part of the primer can be complementary paired with the sequence at the corresponding vector enzyme cut site, the underline is the enzyme cut site.All primers were designed by PRIMER PREMIER 5.0 software. All sequences were sourced from whole genome and dual-seq database . All the accession numbers were from GeneBank. The accession number for *A. phaeospermum* whole genome information in the GenBank database is QYRS0000000.1 . The accession number for dual-seq of *B. pervariabilis* × *D. grandis* infected by *A.phaeospermum* in the NCBI database is SAMN19312317.

Table S2: Information on candidate reciprocal proteins obtained from yeast library screening using *ApCtf1β* as a bait protein

Genebank	Species	Function annotation
XP_010237391.1	*Brachypodium distachyon*	agglutinin isolectin 3
RLN39548.1	*Panicum miliaceum*	peroxidase A2-like
XP_006650917.1	*Oryza brachyantha*	PREDICTED: PAX3- and PAX7-binding protein 1
XP_020171383.1	*Aegilops tauschii subsp. tauschii*	Bowman-Birk type bran trypsin inhibitor-like
TVU43806.1	*Eragrostis curvula*	hypothetical protein EJB05_10301, partial
XP_003557807.1	*Brachypodium distachyon*	non-specific lipid transfer protein-like 1
VAI58483.1	*Triticum turgidum subsp. durum*	unnamed protein product
XP_004958744.1	*Setaria italica*	galactinol synthase 2
KAF0923272.1	*Oryza meyeriana var. granulata*	hypothetical protein E2562_005236
VAH71410.1	*Triticum turgidum subsp. durum*	unnamed protein product
ACF86810.1	*Zea mays*	unknown
KAF2950718.1	*Oryza sativa Japonica Group*	hypothetical protein DAI22_01g207748
AGY80454.1	*Dendrocalamus latiflorus*	ubiquitin-conjugating enzyme
EAY90896.1	*Oryza sativa Indica Group*	hypothetical protein OsI_12510
BAT05803.1	*Oryza sativa Japonica Group*	Os08g0466800
VAI79401.1	*Triticum turgidum subsp. durum*	unnamed protein product
ASU91350.1	*Indosasa hispida*	phenylalanine ammonia lyase
ACI46151.1	*Hordeum vulgare*	NADP-dependent malic enzyme
XP_015694098.1	*Oryza brachyantha*	PREDICTED: uncharacterized WD repeat-containing protein C2A9.03-like isoform X2
AIA26165.1	*Fargesia nitida*	shikimate kinase-like protein 1, partial
AAR18735.1	*Bambusa oldhamii*	chitinase
TKW04132.1	*Setaria viridis*	hypothetical protein SEVIR_7G089300v2
KAF0927229.1	*Oryza meyeriana var. granulata*	hypothetical protein E2562_031026
XP_006649725.1	*Oryza brachyantha*	PREDICTED: protein SPA, chloroplastic
XP_003557727.1	*Brachypodium distachyon*	ATP-dependent Clp protease proteolytic subunit 6, chloroplastic
NP_001152176.1	*Zea mays*	uncharacterized protein

		LOC100285814
KAF0924517.1	*Oryza meyeriana var. granulata*	hypothetical protein E2562_010164
XP_003569184.1	*Brachypodium distachyon*	B2 protein
VAH93822.1	*Triticum turgidum subsp. durum*	unnamed protein product
KAF0905818.1	*Oryza meyeriana var. granulata*	hypothetical protein E2562_008868
XP_025814151.1	*Panicum hallii*	GDSL esterase/lipase At5g45920-like
XP_025819944.1	*Panicum hallii*	26S proteasome non-ATPase regulatory subunit 8 homolog A

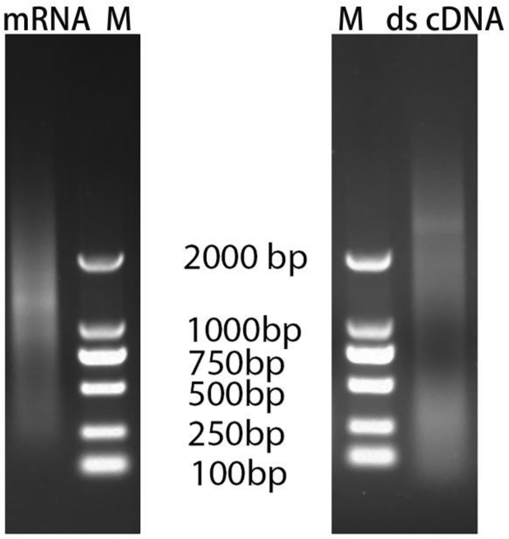

Figure S1. *B.pervariabilis*×*D.grandi* mRNA and ds cDNA electrophoresis assay. M:DL2000 DNA Marker

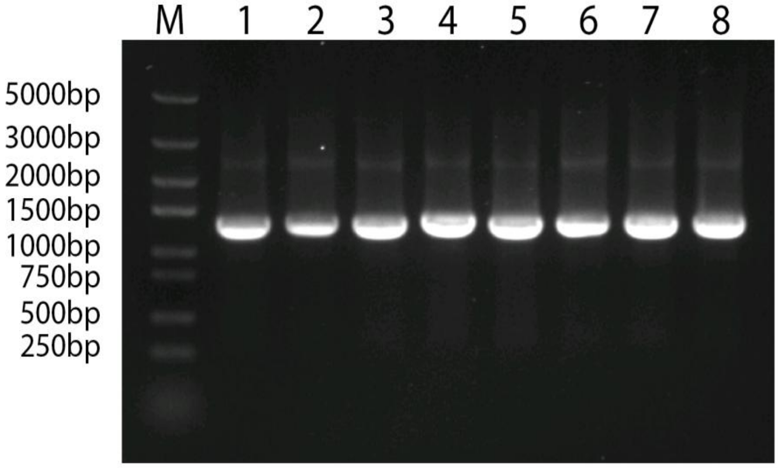

Figure S2. PCR detection of recombinant vector pGBKT7-*ApCtf1β* bacteriophage. DL5000 DNA Marker, 1~8: recombinant plasmids

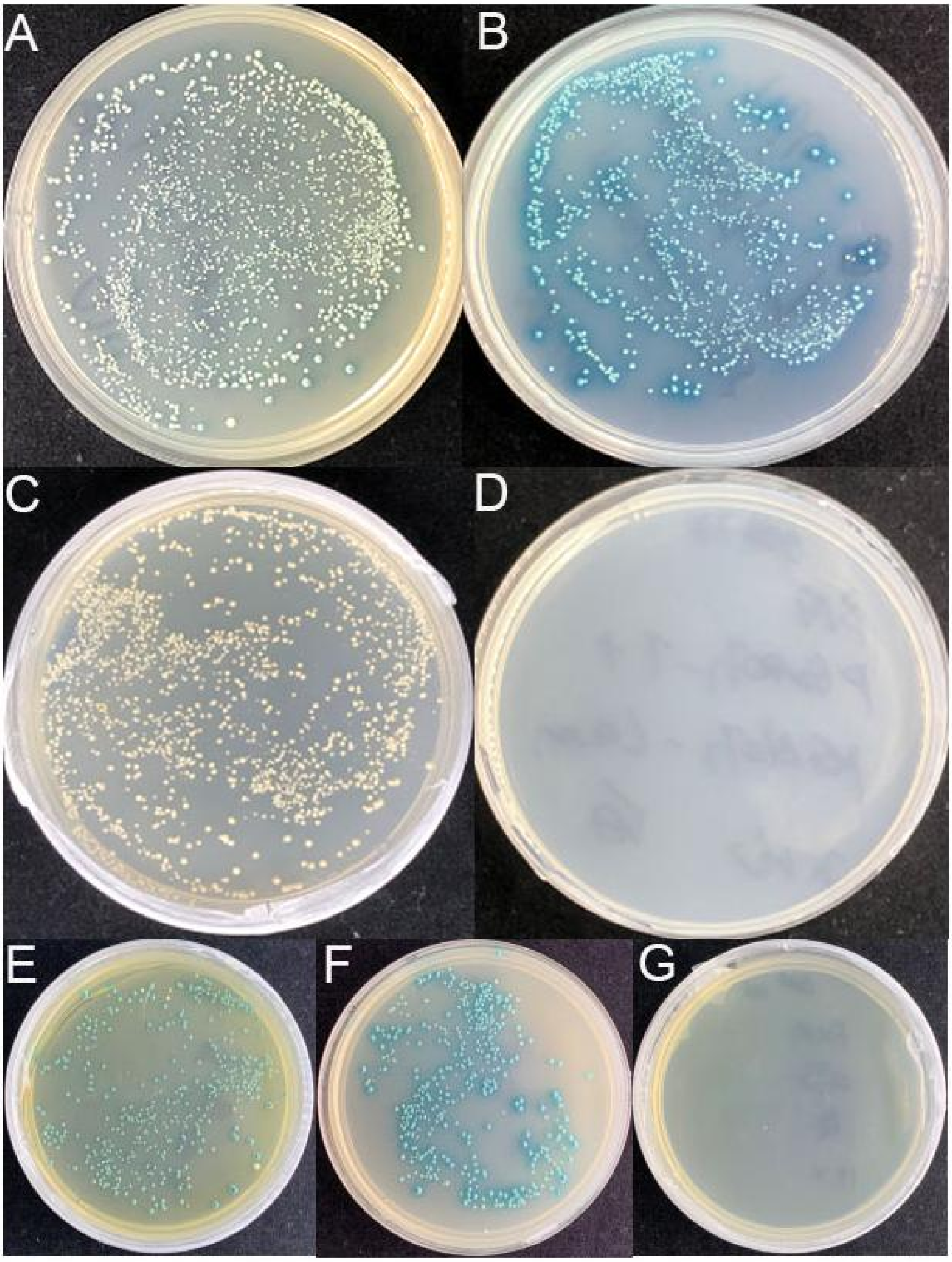

Figure S3. Transcriptional self-activation and toxicity assay of bait vectors.(A): pGBKT7-53 and pGADT7-T cotransformed onto DDO/X medium;(B): pGBKT7-53 and pGADT7-T cotransformed onto QDO/X/A medium;(C): pGBKT7-Lam and pGADT7-T cotransformed onto DDO/X medium;(D): pGBKT7-Lam and pGADT7-T cotransformed onto on QDO/X/A medium;(E): pGBKT7-*ApCtf1β* and pGADT7 cotransformed onto DDO/X medium;(F): pGBKT7-*ApCtf1β* and pGADT7 cotransformed onto TDO/X medium;(G): pGBKT7-*ApCtf1β* and pGADT7 cotransformed onto QDO/X/A medium.

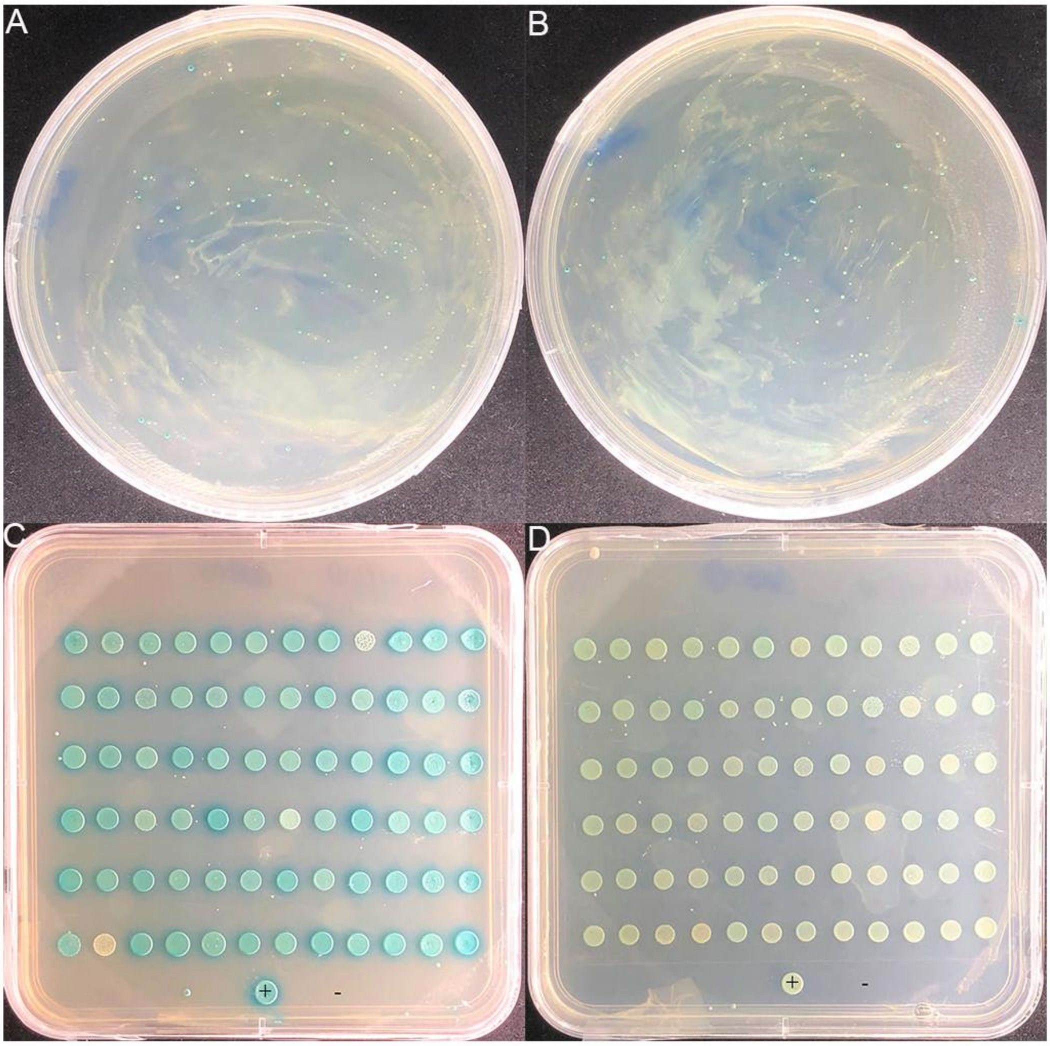

Figure S4. Decoy vector sieve library, (A),(B): pGBKT7-*ApCtf1β* and pGADT7 libraries co-transformed onto QDO/X/A medium for primary screening ;(C),(D): pGBKT7-*ApCtf1β* and pGADT7 libraries co-transformed onto QDO/X/A medium for re-screening; +: positive control; -: negative control.

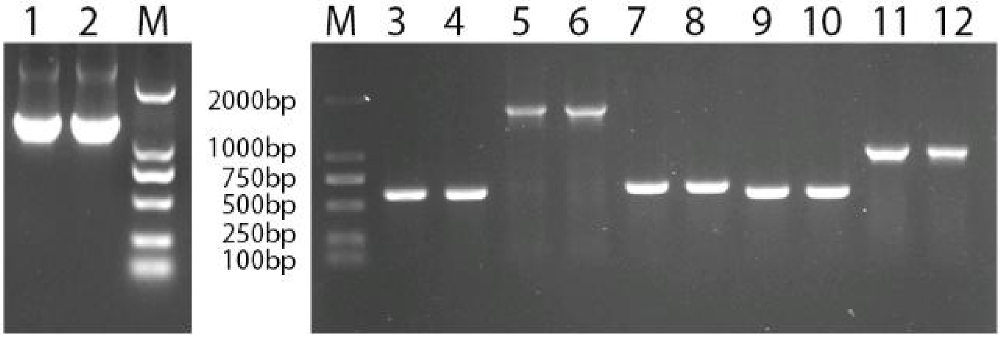

Figure S5. BiFC recombinant plasmids. M : DL2000 DNA Marker;1,2: pSPYNE(R)173-*ApCtf1β*;3,4: pSPYCE(M)-*BDUbc*;5,6: pSPYCE(M)-*BDNADP-M*; 7,8: pSPYCE(M)-*BDWGA-3*;9,10: pSPYCE(M)-*BDSKL1*; 11,12: pSPYCE(M)-*BDGolS2*.

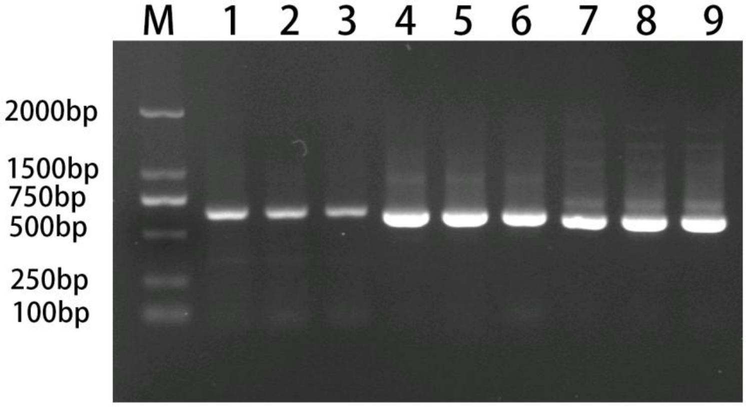

Figure S6. PCR assay of recombinant vector bacterial solution. M:DL2000 DNA Marker; 1~3: PGEX-6P-1-*ApCtf1β* recombinant vector; 4~5: pET28a-*BDUbc*; 7~9: pET28a-*BDSKL1*.

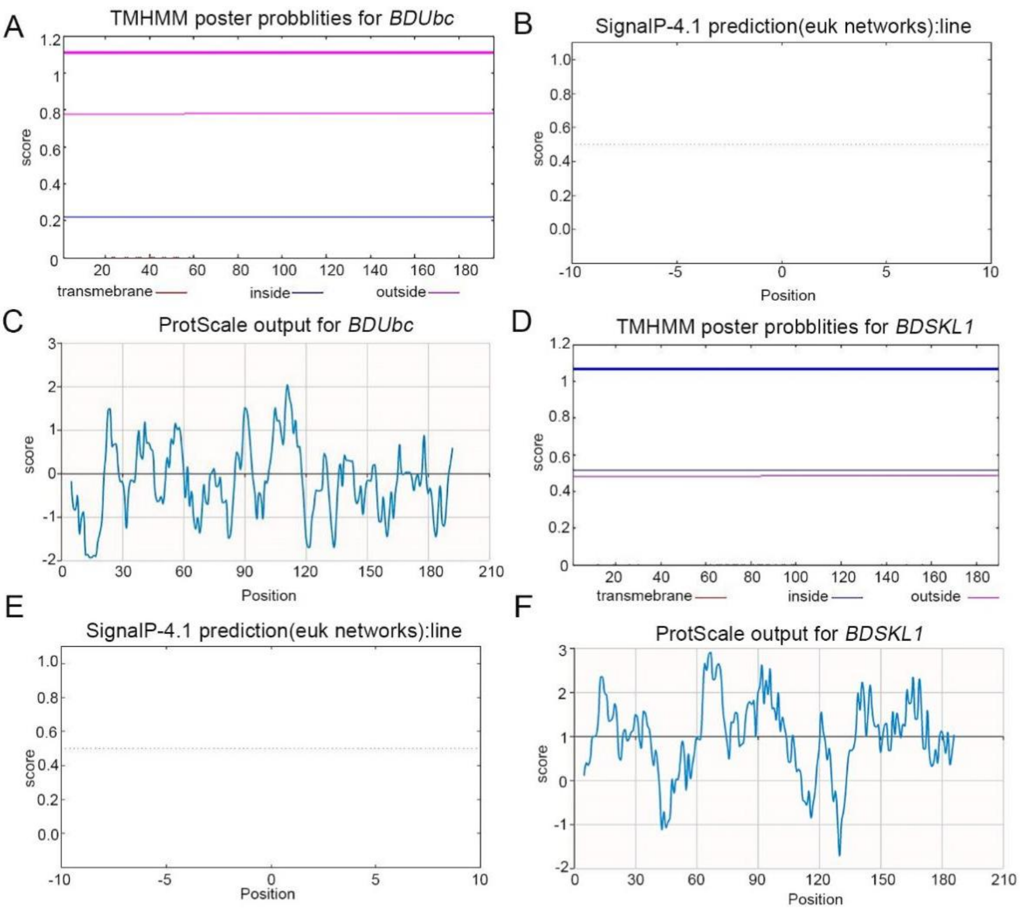

Figure S7. Transmembrane region, signal peptide, hydrophobicity of *BDUbc* and *BDSKL1*.(A),(D): transmembrane region prediction of *BDUbc* and *BDSKL1*;(B),(E): signal peptide prediction of *BDUbc* and *BDSKL1*;(C),(F): hydrophobicity prediction of *BDUbc* and *BDSKL1*.

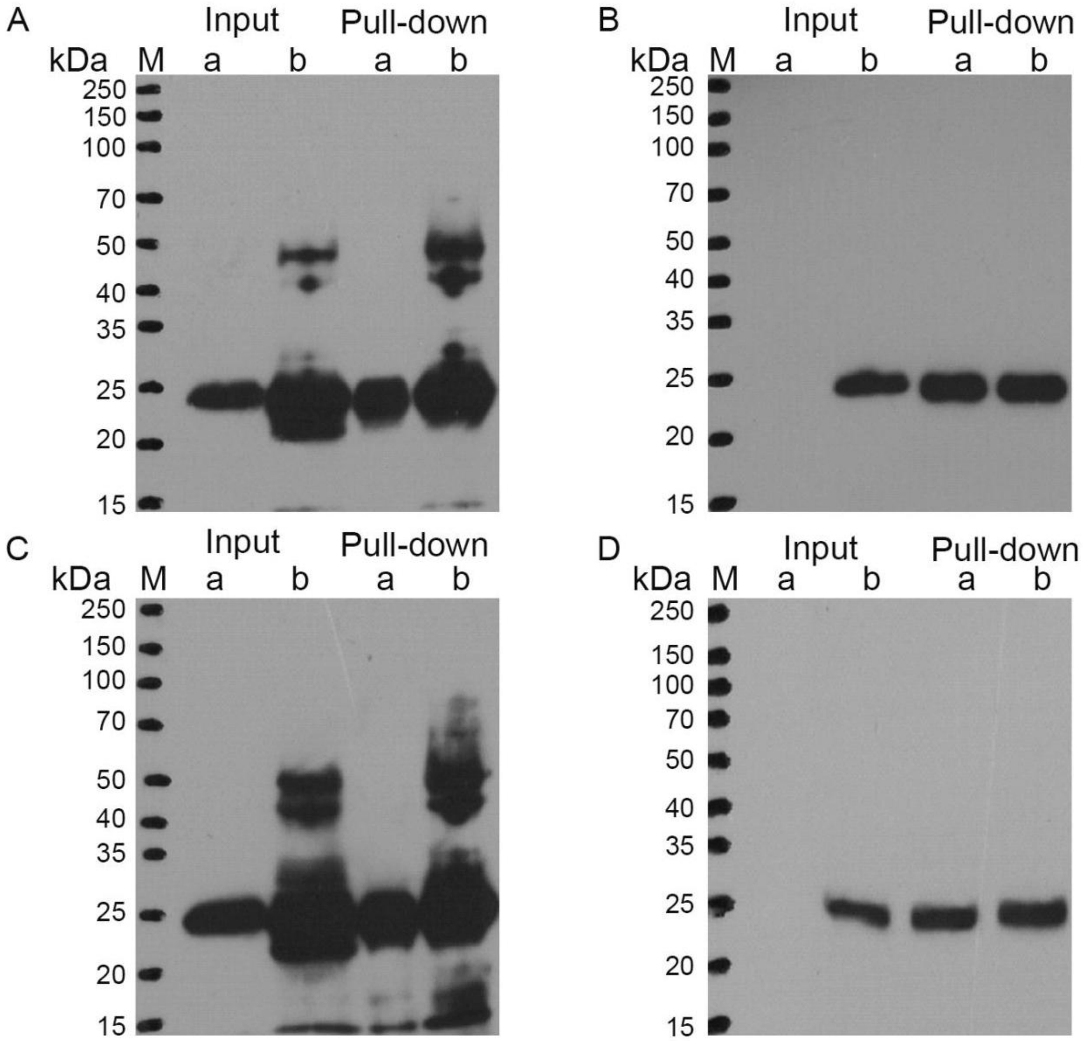

Figure S8. (A) Detect GST tags after pull-down(GST-*ApCtf1β* and His-*BDUbc*) ; (B): Detect HIS tags after pull-down(GST-*ApCtf1β* and His-*BDUbc*); (C) Detect GST tags after pull-down (GST-*ApCtf1β* and His-*BDSKL1*); (D): Detect HIS tags after pull-down(GST-*ApCtf1β* and His-*BDSKL1*); a: control group; b: experimental group.

Table S3. Expression stability of ten reference genes analyzed by GeNorm, Normfinder and BestKeeper

GeNorm			NormFinder			BestKeeper		
Rank	Gene	M value	Rank	Gene	Stabel value	Rank	Gene	Standard deviation
1	*GAPDH*	0.165	1	*Actin*	0.048	1	*GAPDH*	0.275
1	*Actin*	0.165	2	*GAPDH*	0.077	2	*Actin*	0.285
3	*EF1α*	0.233	3	*PP2A*	0.096	3	*RPL3*	0.346
4	*PP2A*	0.263	4	*RPL3*	0.128	4	*EF1α*	0.374
5	*RPL3*	0.335	5	*EF1α*	0.229	5	*PP2A*	0.405
6	*TIP41*	0.445	6	*TIP41*	0.315	6	*TIP41*	0.887

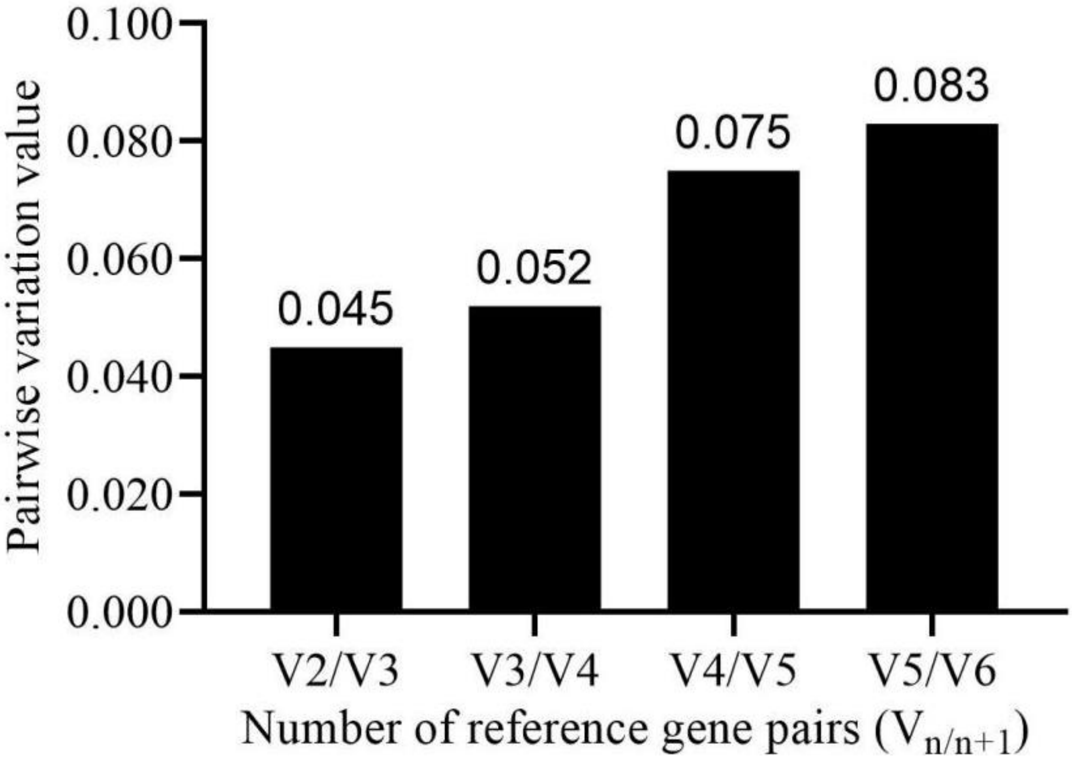

FigureS8. Pairwise variation ($V_{n/n+1}$) analysis of the candidate reference genes.
